# Ambipolar Organic–Inorganic Heterostructure Transistor Array for Integrated Visual Information Processing

**DOI:** 10.1002/advs.75246

**Published:** 2026-04-10

**Authors:** Wen‐Min Zhong, Wenbin Zhang, Yu‐Xiang Zeng, JiYu Zhao, Ziqi Jia, Loganathan Veeramuthu, Guanglong Ding, Yan Yan, Meng Zhang, Su‐Ting Han, Vellaisamy A. L. Roy, Fengyun Wang, Chi‐Ching Kuo, Ye Zhou

**Affiliations:** ^1^ College of Civil and Transportation Engineering Shenzhen University Shenzhen P. R. China; ^2^ Institute for Advanced Study Shenzhen University Shenzhen P. R. China; ^3^ State Key Laboratory of Fine Chemicals Frontiers Science Center For Smart Materials Dalian University of Technology Dalian P. R. China; ^4^ Institute of Organic and Polymeric Materials National Taipei University of Technology Taipei Taiwan; ^5^ State Key Laboratory of Radio Frequency Heterogeneous Integration Shenzhen University Shenzhen P. R. China; ^6^ Department of Applied Biology and Chemical Technology The Hong Kong Polytechnic University Hong Kong P. R. China; ^7^ School of Science and Technology School of Science and Technology Hong Kong Metropolitan University Hong Kong P. R. China; ^8^ Hong Kong Metropolitan University Shenzhen Research Institute Shenzhen P. R. China; ^9^ College of Physics Qingdao University Qingdao P. R. China; ^10^ Advanced Research Center for Green Materials Science and Technology National Taiwan University Taipei Taiwan

**Keywords:** ambipolar transistor, heterostructure, neuromorphic computing, synaptic device, visual information processing

## Abstract

The rapid evolution of artificial intelligence presents not only unprecedented opportunities but also significant technical challenges, particularly in the development of next‐generation computing hardware. To overcome these hurdles, there is an urgent demand for novel chip architectures that offer both ultralow power consumption and high computational efficiency. Neuromorphic computing, inspired by the neural architecture of the human brain, represents a paradigm shift beyond the conventional von Neumann framework, promising remarkable gains in processing capability. Here, we report an ambipolar transistor array based on a vertically stacked polymer/oxide heterostructure, meticulously engineered to integrate electrical computation with optical sensing within a single device. This transistor enables simultaneous electrical and optical modulation, supporting both synaptic transmission under electrical stimuli and dynamic visual information processing under optical inputs. The integrated array system demonstrates efficient and low‐power execution of visual processing, classification, and prediction tasks, highlighting its potential for neuromorphic computing applications such as real‐time traffic analysis. Our findings pave the way for multifunctional and energy‐efficient neuromorphic hardware capable of bridging the gap between sensing and computation.

## Graphical abstract

We present an organic–inorganic heterostructure transistor array for neuromorphic computing, achieving 95.6% MNIST accuracy and 1.2 fJ per operation, with dynamic spatiotemporal encoding and precise vehicle direction detection under combined optical and electrical stimulation. This work paves the way for advanced intelligent sensing and computing applications.

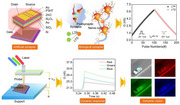

## 1 Introduction

In recent years, machine learning (ML) has been widely deployed to enable various intelligent applications, from decision‐making to content generation [1, 2, 3, 4, 5, 6, 7, 8]. ML encompasses a set of techniques that allow computer systems to perform recognition and prediction tasks through data‐driven learning, rather than explicit programming [9, 10, 11, 12]. The exponential growth in model complexity has intensified the energy efficiency bottleneck of conventional hardware, leading to unsustainable increases in power consumption. In response, the industry is exploring transformative solutions. One promising direction is neuromorphic computing, which draws inspiration from the structure and information processing of biological neural systems. This advanced approach builds asynchronous, event‐driven systems using bionic synaptic devices and spiking neurons [13, 14, 15, 16, 17, 18].

Neuromorphic computing systems demonstrate remarkable capabilities in visual processing and memory. By mimicking the biological integration of perception and memory, this computational architecture enables highly efficient observation and computation. As a result, advancing its on‐chip integration of multimodal sensing and memory has emerged as a key research focus in the field [19, 20, 21, 22, 23]. From a material and device perspective, there is a striking structural analogy between the components of a transistor, such as the source, channel, and drain, and the elements of a biological synapse, including the presynaptic membrane, synaptic cleft, and postsynaptic membrane [24, 25, 26, 27]. The device's unique hole‐electron pair synergistic conduction mechanism offers a physical basis for implementing visual sensing and memory functions [28, 29]. Under external stimuli such as light pulses or electrical signals, the generation, transport, and recombination of charge carriers within the device emulate the release and reuptake of neurotransmitters in biological synapses, allowing dynamic responses to varying inputs [30, 31]. This behavior enables accurate simulation of core synaptic and neuronal functions. Thanks to these synapse‐like attributes, such devices can theoretically act as both synaptic weight regulators and visual perception neurons, presenting a promising pathway toward building efficient neuromorphic vision systems.

**FIGURE 1:**
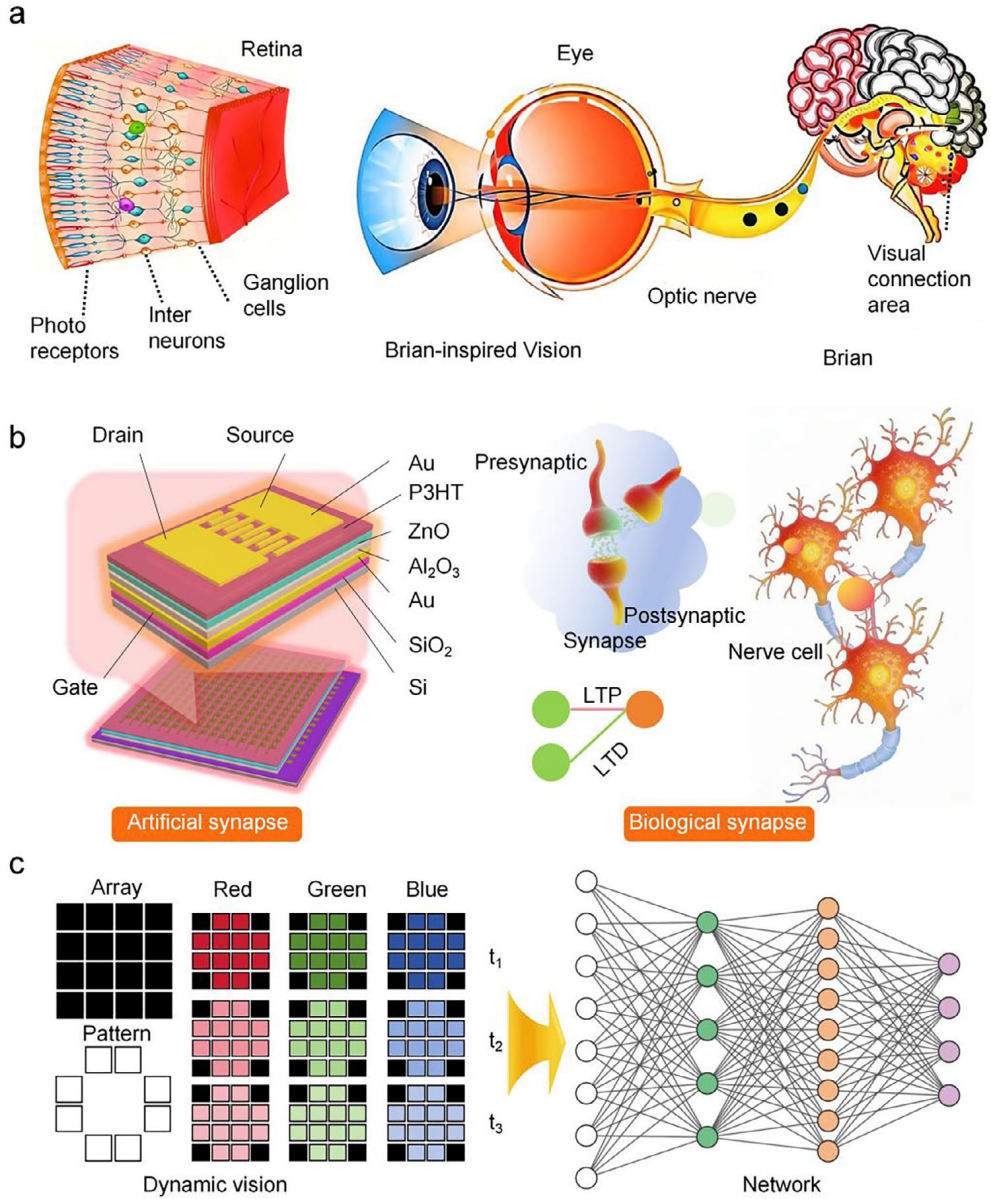
Bio‐inspired principle and system architecture. (a) Diagram of the human eye's visual system. (b) Illustration of the human brain and synaptic neurons. (c) Schematic of a hardware neural network based on these devices for dynamic visual tasks.

Inspired by the efficient visual processing mechanism of the human eye, we propose an optoelectronic neuromorphic computing architecture that integrates front‐end perception with in‐memory computation. As illustrated in Figure 1a, human color vision originates from only three types of cone cells, which function as miniature spectrometers to convert light into electrical signals. These signals are subsequently processed in the visual cortex through additive and subtractive combinations, enabling the perception of over ten million colors. More profoundly, the brain's ∼86 billion neurons and ∼100 trillion synapses compress visual information by over 90% while preserving essential features, effectively condensing temporal sequences into sparse, information‐rich representations. To replicate this feature, we have developed an ambipolar transistor array based on a ZnO/poly(3‐hexylthiophene‐2,5‐diyl) (P3HT) heterojunction, as shown in Figure 1b. This device uniquely combines the electrical programmability of a synaptic transistor with the intrinsic visual adaptation of a photoresponsive material. As an n‐type semiconductor [32, 33, 34], ZnO enables effective electron transport under electrical modulation, while p‐type P3HT [35, 36, 37] exhibits exceptional dynamic photoresponse across the visible spectrum [38, 39, 40]. The synergistic interaction between these materials enables versatile neuromorphic functionalities within a single device. When configured as an optical array unit, the device exhibits a dynamic photoresponse that inherently retains historical stimulus information, effectively encoding temporal visual patterns. When employed as an electrical array unit, it emulates biological synapses by modulating synaptic weights via gate bias, enabling decision‐making and learning. The seamless fusion of these capabilities within a single platform allows optical information to be processed by the electrical array, establishing a fully integrated optoelectronic neuromorphic computing system for dynamic visual processing at the sensory level, as shown in Figure 1c. In‐sensor computing utilizes optically‐induced long‐term memory as synaptic weights for neuromorphic image processing, whereas reservoir computing employs dynamic responses for temporal tasks. This architecture represents a synergistic fusion of both paradigms, specifically designed to process image sequences with inherent temporal characteristics. Rather than handling individual static frames, this approach treats the temporal evolution across all frames as the fundamental object of computation.

## 2 Result and Discussion

**FIGURE 2:**
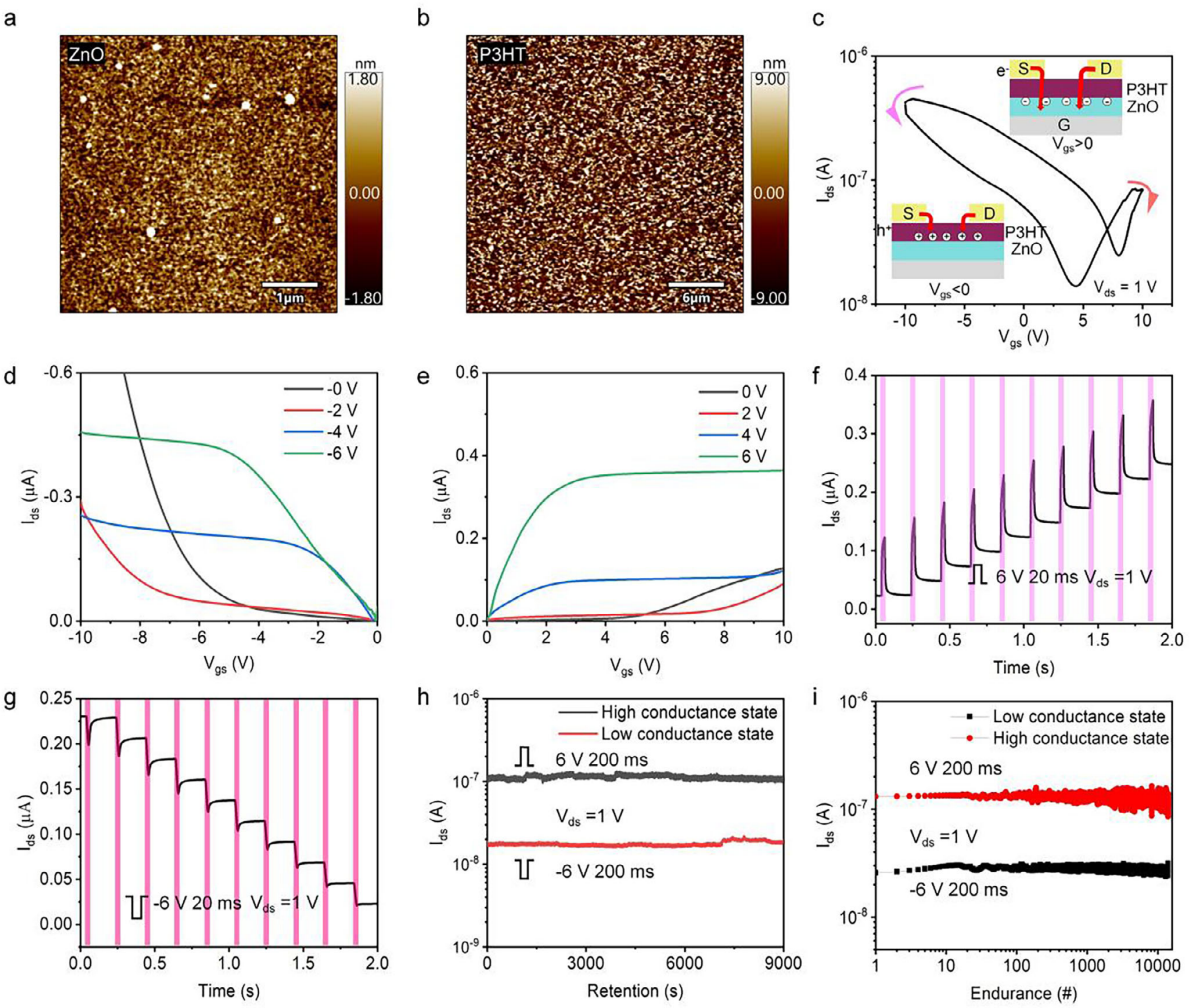
Device structure and electrical performance. (a) Atomic force microscopy images of the ZnO. (b) P3HT layers. (c) Transfer characteristics of the transistor. (d) Output characteristics of the transistor under negative gate voltages. (e) under positive gate voltages. (f) The I_ds_ of device applied positive gate voltage pulses. (g) The I_ds_ of device applied negative gate voltage pulses. (h), Endurance performance of the device. (i) Retention performance of the device.

The transistor structure is composed of a P3HT layer deposited on top of ZnO, which is itself layered on Al_2_O_3_. The Al_2_O_3_ film serves as the insulating layer, while ZnO and P3HT function as n‐type and p‐type semiconductors, respectively. Gold electrodes are thermally evaporated onto the substrate and the P3HT surface to form the gate and source/drain contacts, ensuring efficient electrical interfacing within the device. Figure 2a,b presents the atomic force microscopy (AFM) images revealing the microstructural morphology of the ZnO and P3HT layers, which play a critical role in governing carrier transport. The measured root‐mean‐square surface roughness values are 0.78 nm for ZnO and 7.34 nm for P3HT. By applying a gate voltage, the electron and hole concentrations in ZnO and P3HT can be precisely modulated, enabling the device to exhibit ambipolar operation. Figure 2c displays the transfer characteristics of the transistor, obtained by sweeping the gate voltage (V_gs_) from −6 to +6 V under a fixed drain bias of 1 V. Under positive gate bias, electrons accumulate in the ZnO channel near the dielectric interface, while under negative gate bias, holes accumulate in the P3HT layer. A noticeable hysteresis is observed in the transfer curve, indicating a memory effect that is essential for emulating synaptic behavior. Figure 2d,e shows the output characteristics of the transistor under various gate voltages. The output current increases linearly at low drain voltages and saturates at higher biases, consistent with typical ambipolar transistor operation [41]. The dynamic response of the device under pulsed excitation is illustrated in Figure 2f,g. In the initial state, without any gate bias, the drain current (I_ds_) remains nearly constant. Upon application of a +6 V gate pulse, I_ds_ rises abruptly and then decays nonlinearly, stabilizing above the initial baseline. This behavior resembles synaptic excitation, where residual charge carriers in the channel enhance conductivity after a stimulus. Repeated positive pulses further increase channel carrier density, progressively strengthening the conductance, which is a form of synaptic potentiation [42, 43]. Conversely, negative gate pulses induce synaptic depression by depleting carriers. By modulating the gate with voltage pulses, we successfully emulate synaptic excitation and inhibition, enabling the implementation of neuromorphic computing paradigms for judgment and decision‐making tasks. To assess reliability, endurance, and retention tests were conducted. As shown in Figure 2h,i, the device maintains stable resistance states over 10^4^ s and withstands more than 10^5^ pulse cycles, demonstrating excellent retention and fatigue resistance.

**FIGURE 3:**
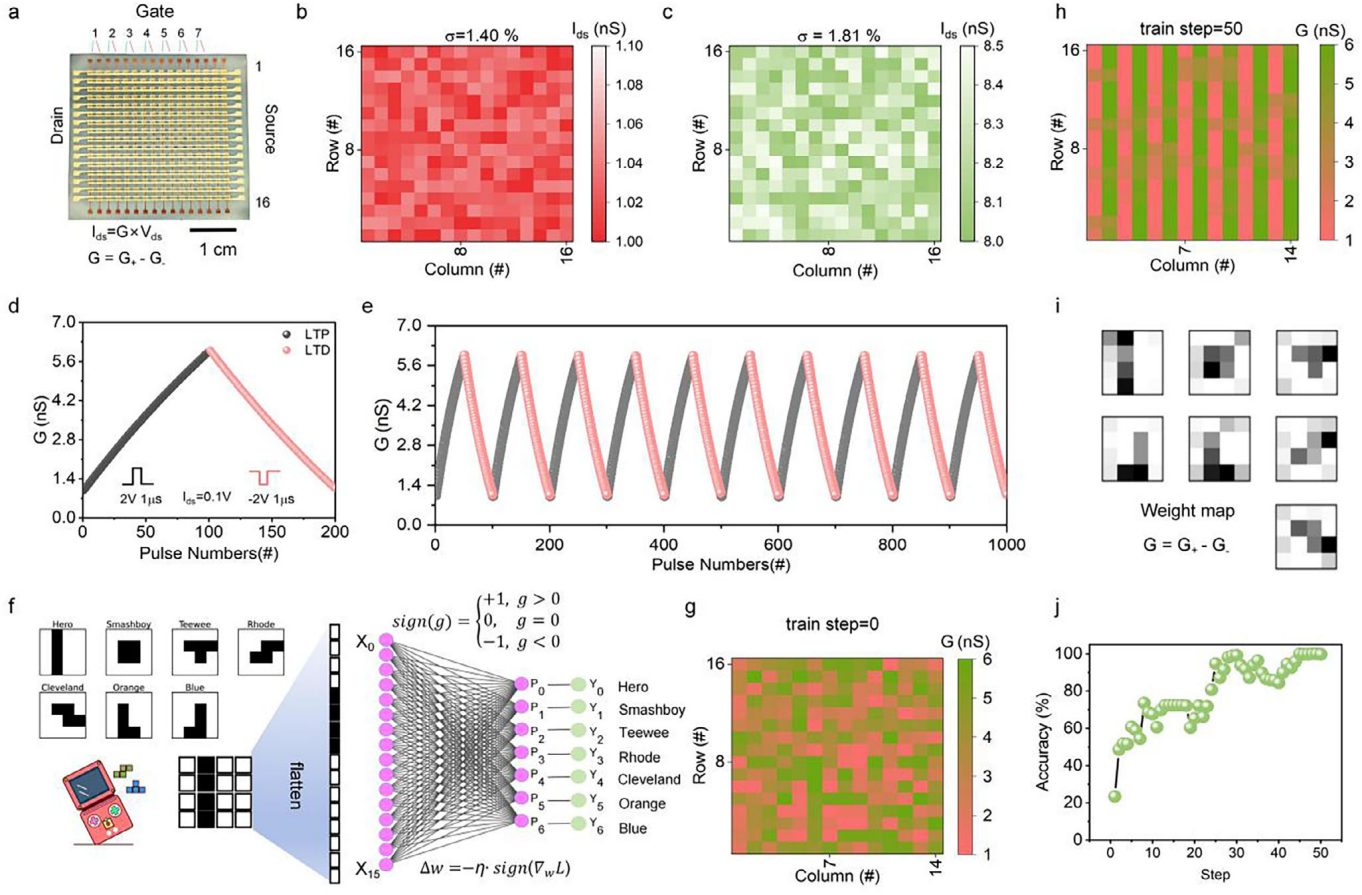
Array integration and neuromorphic computing applications. (a) Optical microscopy image of the 16 × 16 transistor array. (b) Conductance distribution mapping of the array at low conductance state. (c) at high conductance state. (d) Long‐term potentiation and long‐term depression characteristics achieved through pulse modulation, demonstrating multi‐state conductance tuning. (e) Repeatability test over 10 LTP/LTD cycles. (f) Schematic of the Tetris pattern recognition task using the array. (g) conductance state distribution before training. (h) conductance state distribution after 50 training epochs. (i) Synaptic weight mapping represented by the conductance difference after training. (j) Recognition accuracy over 50 training epochs for the Tetris task.

The fabricated 16 × 16 transistor array consists of 256 individual units, serving as a compact processor for recognition tasks. Figure 3a shows the optical image of the integrated array. Following the previously established approach, synaptic excitation and inhibition were emulated using positive and negative voltage pulses, respectively. Prior to recognition tasks, the array was preconditioned with +6 and −6 V pulses of 500 ms duration to assess device uniformity. The array employs ±6 V, 500 ms pulses for initialization operations to restore the devices to their baseline states, and utilizes ±2 V operating voltage for weight update operations to modulate synaptic states. Figure 3b,c displays the conductance distributions under the low‐conductance state (LCS) and high‐conductance state (HCS), respectively. Statistical analysis yielded coefficient of variation (σ) values of 1.40% for LCS and 1.81% for HCS, indicating satisfactory uniformity for neuromorphic operation. Subsequently, the array was driven with smaller voltage amplitudes (±2 V) and shorter pulse widths (1 µs) to perform recognition tasks. This approach enables continuous modulation of device conductance into multiple intermediate levels, facilitating fine‐grained control of synaptic excitation and inhibition [44, 45]. As shown in Figure 3d, continuous +2 V stimulation progressively increases the conductance, while −2 V stimulation leads to a gradual decrease. The conductance rises from approximately 1 to 7 nS under repeated positive pulses and recovers to the baseline under negative pulses, effectively mimicking long‐term potentiation (LTP) and long‐term depression (LTD) behaviors. This reversible modulation reflects a biologically plausible learning and forgetting mechanism [46, 47]. The device exhibits low nonlinearity in both LTP (Np = 0.003) and LTD (Nd = 0.004), as shown in Figure S1a. Notably, nonlinearity is voltage‐dependent, with significantly degraded values at 4.5 V (Np = 0.056, Nd = 0.053), highlighting the advantage of low‐voltage operation. The array consumes only 1.2 fJ per synaptic event and achieves 95.6% accuracy on the MNIST dataset using a convolutional neural network (Figure S2), a performance competitive with existing neuromorphic platforms. A comparative summary of recent neuromorphic devices in terms of power consumption and MNIST accuracy is provided in Table S1. Figure 3e shows the conductance response over ten consecutive LTP/LTD cycles, demonstrating high stability and repeatability. To validate the array's learning capability, we constructed a recognition task based on Tetris patterns, which can be represented as 4 × 4 pixel images spanning seven categories. Figure 3f illustrates the schematic of the Tetris pattern recognition setup. Each 4 × 4 image was flattened and converted into voltage signals, which were applied to the drain terminals of the transistor array. Gate voltage pulses were then sequentially applied to implement weight updates in a single‐layer perceptron configuration. Using a differential mapping scheme, synaptic weights were represented by the conductance difference between adjacent ambipolar devices (denoted as G^+^ and G^−^), with the effective weight defined as G = G^+^ – G^−^ [45, 46, 47]. Initially, the processor exhibited random weight distribution due to its untrained state, as shown in Figure 3g. After 50 training cycles, the weights evolved into structured patterns (Figure 3h), and analysis of the weight matrix G revealed a clear mapping to the Tetris patterns, indicating that distinct device regions specialize in recognizing different shapes (Figure 3i). The recognition accuracy improved from an initial 14% to 100% after training, confirming successful learning, as summarized in Figure 3j.

**FIGURE 4:**
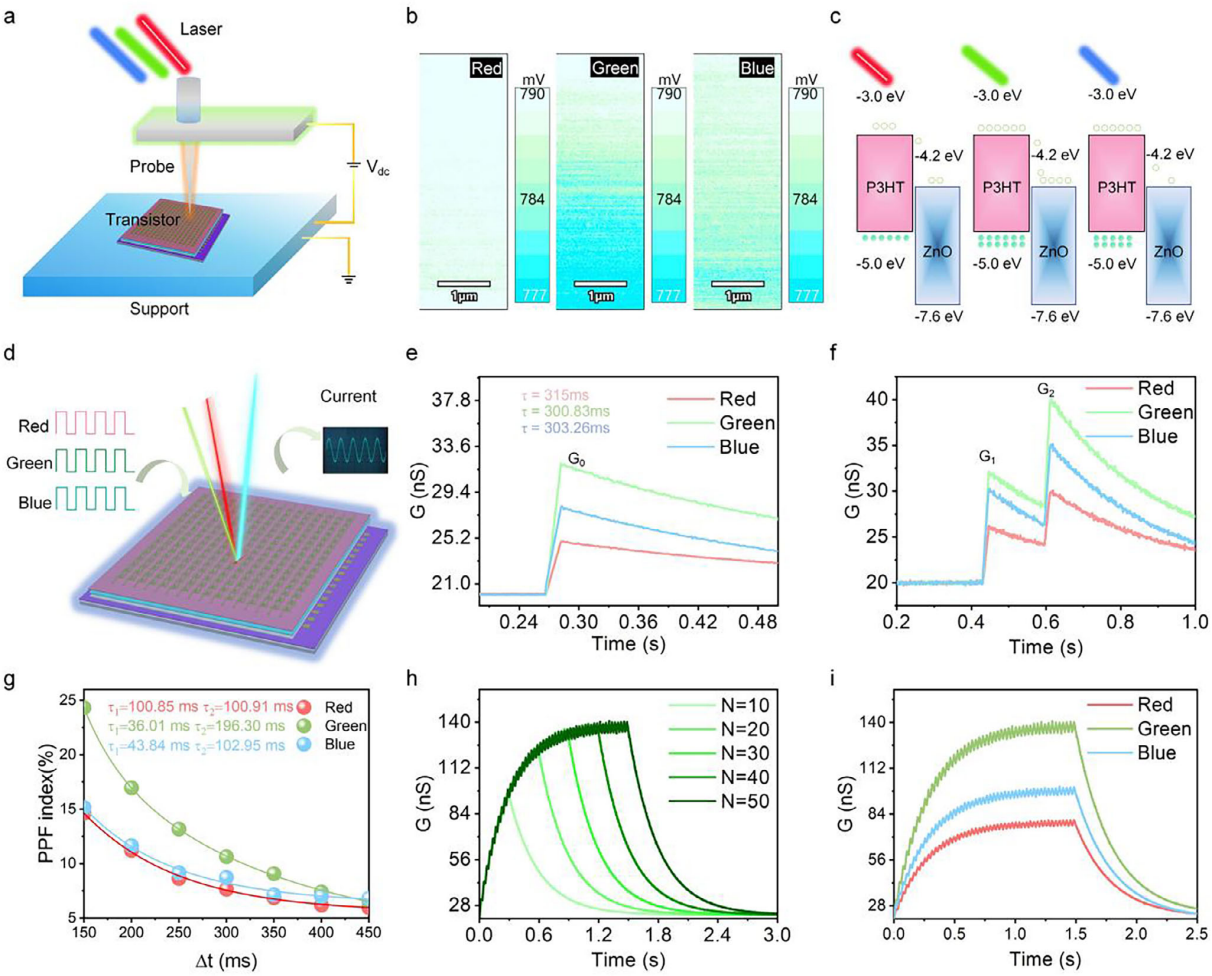
Photoresponse properties and dynamic characteristics. (a) Schematic of the Kelvin probe force microscopy measurement. (b) KPFM surface potential images of the P3HT film under red, green, and blue monochromatic light illumination. (c) Schematic of carrier generation mechanisms under different wavelength illumination. (d) schematic diagram of optoelectronic response test. (e) Short‐term memory performance and decay time constants after a single optical pulse illumination. (f) Paired‐pulse facilitation characteristics. (g) PPF index as a function of pulse interval. (h) Impact of green light pulse number on STM (pulse width = 10 ms). (i) Comparison of device conductance after 50 consecutive pulses of three light colors (pulse width = 10 ms).

Due to the distinct mechanisms of optical and electrical stimulation, the device can achieve stimulus responses at different levels, enabling clear differentiation between the two modes. In order to further understand the device's optoelectronic properties, we conducted a detailed characterization of the heterojunction film. In particular, Kelvin Probe Force Microscopy (KPFM) was employed to evaluate the photoresponse under varying illumination wavelengths. As illustrated in Figure 4a, a conductive atomic force microscopy probe was used to scan the P3HT surface under illumination, measuring the corresponding surface potential changes. The light source comprised tunable LEDs emitting red (625 nm), green (525 nm), and blue (470 nm) light, with a spot diameter of approximately 50 µm and a fixed power density of 0.8 mW cm^−^^2^. Figure 4b presents KPFM images of the same device region under the three monochromatic illuminations. All wavelengths induced a noticeable reduction in surface potential, with the most pronounced decrease observed under green light, consistent with the absorption peak of P3HT around 530 nm. This correlation highlights the direct relationship between the film's absorption coefficient and the yield of photogenerated carriers [48, 49]. The carrier generation mechanisms under different wavelengths are illustrated in Figure 4c. Green light optimally excites π–π* transitions, generating a high density of localized polaron pairs within and between polymer chains. In contrast, red light primarily activates deep trap states due to its lower photon energy, while blue light, despite its higher energy, is limited by shallow penetration, which results in enhanced surface recombination. As a result, the optoelectronic response is strongest under green illumination, moderate under blue, and weakest under red light. This wavelength‐dependent behavior enables the extraction of richer image features through spectral discrimination. Upon pulsed optical stimulation, the device exhibits a transient photoconductive response: conductivity rises instantaneously and then decays nonlinearly to the baseline, demonstrating short‐term dynamic behavior, as shown in Figure 4d. Figure 4e quantifies the short‐term memory (STM) characteristics under each wavelength. The transient response follows an exponential decay, with forgetting times (τ) of 303.26, 300.83, and 315 ms for green, red, and blue light, respectively. The maximum conductance modulation (ΔG/G_0_) under green light was approximately 43.18%, about 2.4 times that of red light (17.84%) and 1.1 times that of blue light (39%), confirming the role of wavelength in controlling photogenerated carrier density [50, 51, 52]. The device also exhibits pair‐pulse facilitation (PPF), a form of short‐term synaptic plasticity. As shown in Figure 4f, when two consecutive green light pulses (10 ms width, 0.8 mW cm^−^^2^) were applied with a Δt = 150 ms interval, the PPF index reached 24.33%, compared to 14.60% and 15.15% for red and blue light, respectively. Figure 4g further summarizes the PPF index as a function of pulse interval. The decay time constants (τ_1_ and τ_2_) extracted from double‐exponential fitting were 100.85 ms/100.91 ms for green light, and 43.84 ms/102.95 ms for both red and blue light, confirming the role of wavelength in modulating temporal dynamics. Notably, these transient responses can be nonlinearly integrated under repeated stimulation. As shown in Figure 4h, the conductance baseline progressively elevates with increasing numbers of green light pulses. After 10 to 50 pulses, the post‐illumination conductance increased from 97.70 to 140.17 nS, though it eventually decayed to the initial state, mimicking the short‐term retention and decay of visual impressions in biological vision. Similar behavior was observed under red and blue illumination (Figure S3). Figure 4i compares the peak conductance after 50 consecutive pulses (10 ms width, 10 ms period) for each wavelength: green light induced the highest conductance (140.17 nS), followed by blue (100.50 nS) and red (80.30 nS). These results demonstrate that the device exhibits distinct, wavelength‐specific perceptual effects, underscoring its capability for dynamic and color‐discriminative optoelectronic response.

**FIGURE 5:**
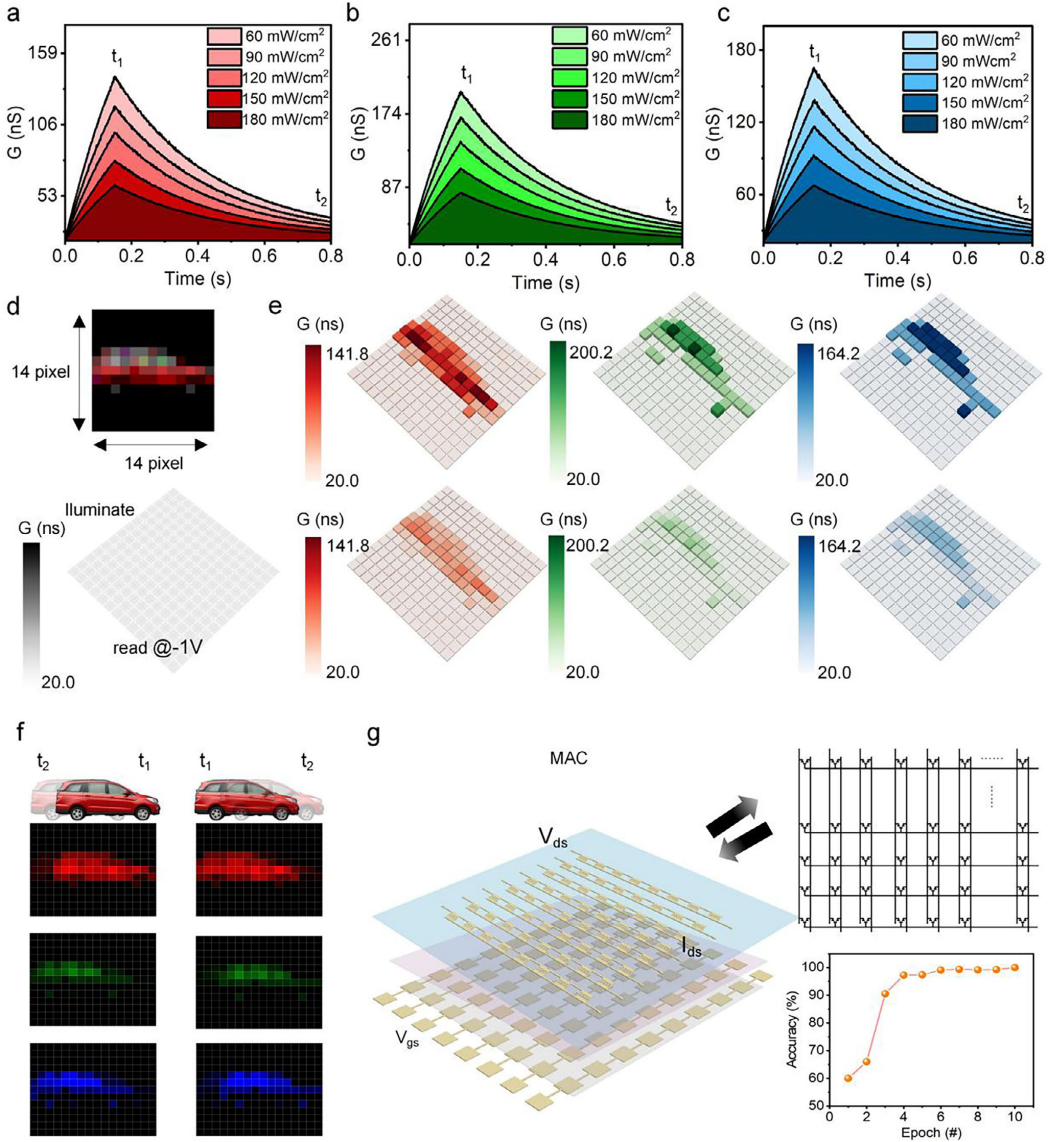
Dynamic visual processing and behavior recognition. (a) Photo‐response of the device under sequentially illuminated red with increasing intensity (60 to 180 mW/cm^2^, pulse width = 150 ms). (b) green light. (c) blue light. (d) 5‐level‐quantized top‐view image of a miniature car and the 16 × 16 transistor array for visual processing. (e) Optoelectronic response and short‐term memory of the array to the car. (f) Dynamic visual effects of the array visualization cart. (g) Optoelectronic combined recognition of car driving behavior task.

The dynamic response of the device can be modulated not only by the number of optical pulses but also by the illumination intensity. As shown in Figure 5a–c, the photocurrent increases progressively as the light intensity rises from 60 to 180 mW cm^−^^2^. Under red light, the peak conductance increases from 61.58 to 141.76 nS; under green light, from 80.59 to 200.18 nS; and under blue light, from 67.57 to 165.48 nS. This intensity‐dependent behavior enables the device to encode color‐rich visual scenes. Taking a car as an example: in real‐world scenarios, object colors are composed of RGB components. As illustrated in Figure 5d, the original color image of a car is decomposed into grayscale values, which are then converted into optical pulses of varying intensities. Owing to the dynamic photoresponse of the device, the conductance does not immediately vanish upon cessation of illumination but persists for a duration until the photogenerated carriers recombine. As a result, temporal sequences can be discerned from the decaying photoresponse. Figure 5e illustrates this behavior: when a car is observed at t_1_ = 0 s and t_2_ = 0.8 s, the three color channels capture corresponding features. After the light source is removed, the photoresponse decays nonlinearly, leaving detectable traces even after 0.8 s. This persistence suggests the device's capability to convey dynamic visual information. The residual signal effectively embeds historical temporal data, allowing both past and current visual information to be represented within a single frame. When a car moves, it leaves a trail of reflections along its path. If the readout time is set to 0.8 s, the array registers reflections of varying intensities, which can serve as references to determine the direction of motion, indicating whether the car is moving forward or backward, as depicted in Figure 5f. This principle readily generalizes to motion along vertical and horizontal directions (up, down, left, right). To systematically evaluate this capability, we constructed an eight‐class dataset comprising 32 × 32 × 3 pixel images, representing a car performing all possible motion combinations: forward/backward translations along the ±x and ±y axes, each in both “drive” and “reverse” modes [53, 54]. The eight categories are: left‐forward, left‐backward, right‐forward, right‐backward, up‐forward, up‐backward, down‐forward, and down‐backward. Each composite image was zero‐padded to 32 × 32 pixels, duplicated across RGB channels, and augmented with 0.1% Gaussian noise (σ = 0.001 relative to full scale). A total of 800 images were generated and used to train a hardware‐emulated convolutional neural network [55, 56, 57]. The network achieved 100% classification accuracy on the test set, demonstrating that the in‐sensor analog weights can reliably discriminate motion directionality solely from color‐specific afterimages, as shown in Figure 5g. These results confirm that the visual array, after hardware neural network training, can support basic behavioral judgment for moving objects such as vehicles.

**FIGURE 6:**
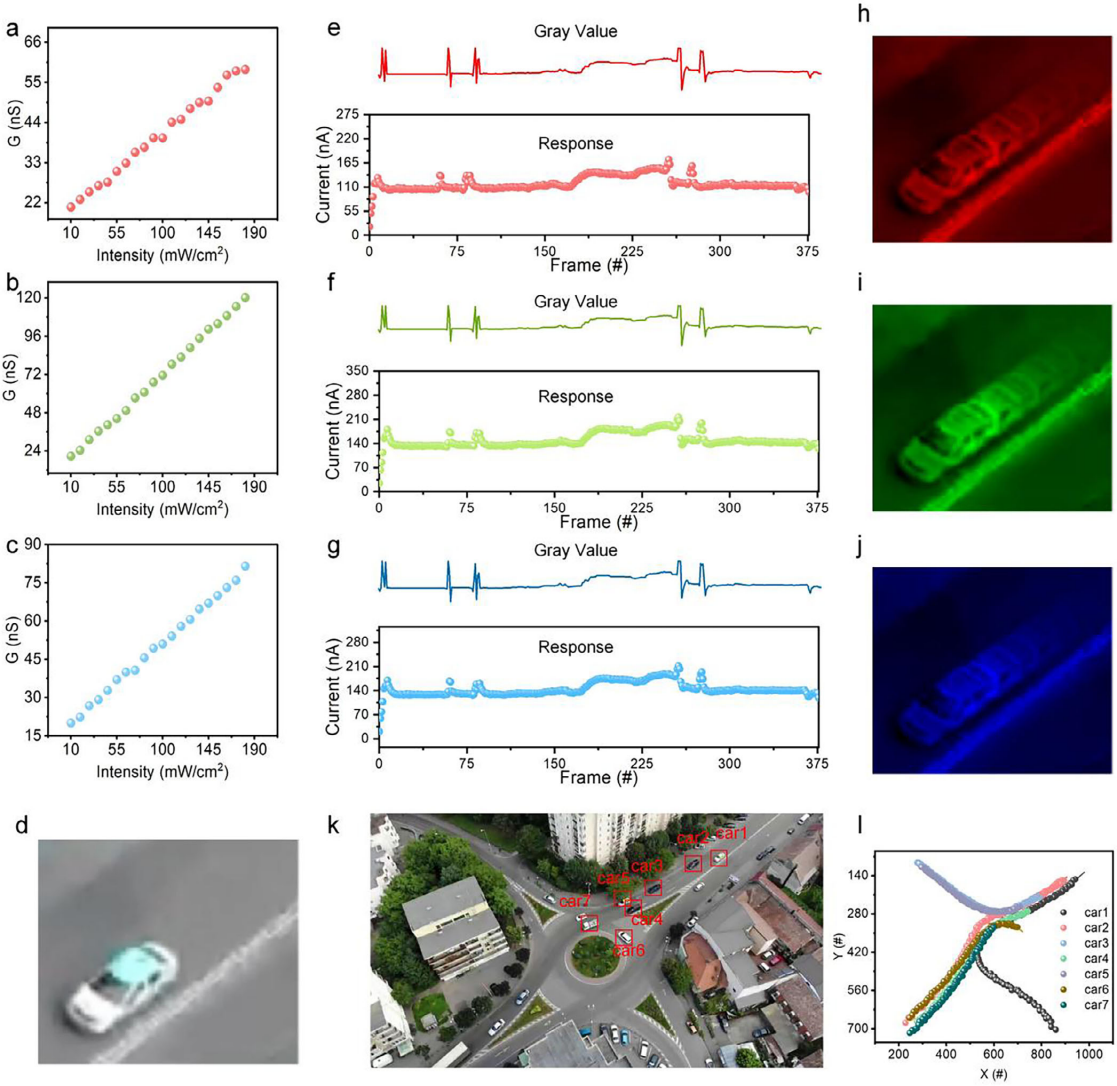
Validation in real‐world traffic scenario and prediction. (a) Optoelectronic response of the device under different light intensity (red irradiation). (b) Green irradiation. (c) Blue irradiation. (d) Video frame of a real‐world vehicle driving scenario. (e) Photo‐response of the device throughout 376 frames of the video (red irradiation). (f) Green irradiation. (g) Blue irradiation. (h) Visualization of the array's photo‐response across Red channel at the 5th frame. (i) Blue channel. (j) Green channels. (k) Real‐time traffic conditions with seven vehicles in different states selected for prediction. (l) Comparison of the actual and predicted trajectories for the seven vehicles.

For complex visual scenarios, the photo‐synapse also exhibits robust responsiveness. Figure 6a–c illustrates the optoelectronic response of the device under varying light intensities. The nearly linear correlation between photocurrent and illumination intensity confirms the device's suitability for processing real‐world visual information. Owing to the intrinsic dynamic response of the device, captured images inherently retain temporal attributes. As an example, Figure 6d displays the first frame of a real‐world traffic video sequence. As the video progresses, individual pixels evolve dynamically due to vehicle motion, resulting in event‐driven changes localized in both space and time. When the optical sensing unit detects such event‐triggered variations, it generates photoresponse patterns that differ from those of the static background. In conventional sensor arrays, the response to optical pulses is largely uniform across pixels. In contrast, our array exhibits a delayed response due to its dynamic charge transport mechanism. As shown in Figure 6e–g, a discernible lag is observed between the optical input and the photoelectric output. This delay, originating from the device's dynamic behavior, effectively embeds historical visual information into the resultant image. Figure 6h–j presents the imaging results of the array in the R, G, and B channels, respectively. Notably, vehicle trajectories that are absent in the original static frame are clearly resolved by the array. These residual traces can serve as a basis for inferring future vehicle behavior. For instance, in the residual image, the trace appears brightest at time t_1_ and darkest at time t_5_. By analyzing the spatial brightness distribution, the direction of motion can be inferred: if the brightest region lies to the left and the dimmest to the right, the vehicle is likely moving leftward. The optoelectronic response G can be spatially mapped as (xG, yG), where (x, y) denotes the real‐time vehicle position. By collecting G values from t_1_ to t_5_, it becomes possible to predict the subsequent position (x_0_, y_0_) at t_0_, as illustrated in Figure S4. We selected seven vehicles and compiled their real‐time location data along with residual photoresponse into a dataset for next‐frame position prediction. Figure 6k shows the seven vehicle samples used. Using a feedforward neural network, we predicted their positions at the next time step. As shown in Figure 6l, the model converged effectively, with predictions aligning closely with ground truth, validating the utility of historically retained photoresponse for motion forecasting. Although various optoelectronic neuromorphic devices have been reported, existing research remains largely confined to static image processing or simple pattern classification, with insufficient attention to temporal information processing and dynamic vision tasks. This tendency decouples spatial from temporal information processing, and a unified spatiotemporal collaborative filtering architecture remains underdeveloped [58, 59].

**FIGURE 7:**
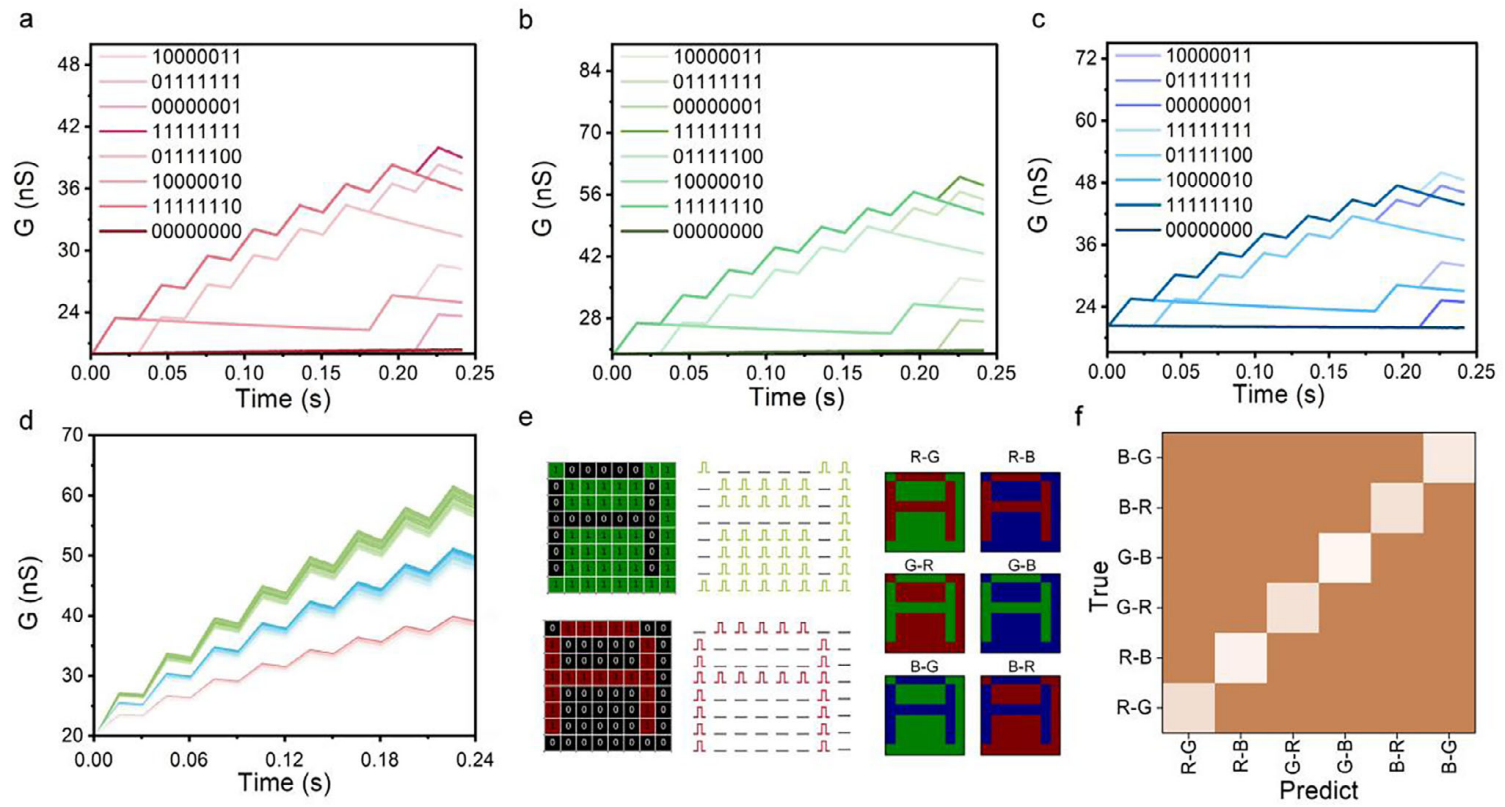
Reservoir computing and color information encoding. (a) Six color variations of an uppercase letter ‘A’ and the 8‐bit pulse encoding scheme using red light irradiation. (b) Using green light irradiation. (c) using blue light irradiation. (d) Stability test results for the code ‘11111111’ over 50 trials. (e) Schematic of the letter ‘A’ with pulse encoding. (f) Confusion matrix for letter recognition using 8‐bit pulse encoding, achieving 100% accuracy.

In addition, the transistor array can function as an optoelectronic reservoir computing unit. Leveraging the dynamic response characteristics of the device, we implemented a partial 8‐bit encoding scheme and designed a color letter recognition task. The encoding time window was set to 15 ms, where “0” represents no optical pulse and “1” corresponds to a 15 ms pulse, followed by a 15 ms relaxation interval. After applying eight consecutive pulses, the inherent nonlinearity of the device enables decoding based on the conductance measured at the final time step. As summarized in Table S2, distinct conductance states are obtained for all possible 8‐bit encoding sequences. Owing to the wavelength‐dependent photoresponse of the device, red, blue, and green light elicit different levels of conductance modulation. This allows the 8‐bit optical encoding to generate a diverse set of analog conductance states, which more richly represent image features compared to conventional binary responses. As shown in Figure 7a–c, the encoding patterns under different illumination colors exhibit similar trends, yet the varying optoelectronic efficiencies lead to differentiated conductance distributions. Overall, the device achieves a 256‐level mapping capability, forming the basis for a color‐graded dataset. We constructed a color‐coded dataset labeled as ‘B‐G’, ‘B‐R’, ‘R‐G’, ‘R‐B’, ‘G‐B’, and ‘G‐R’, where the first letter denotes the color of the letter and the second indicates the background color. To evaluate encoding stability, the sequence ‘11111111’ was tested over 50 repeated trials. Figure 7d confirms the consistency of the pulse‐encoded response, with minimal variation observed across cycles. For visual illustration, Figure 7e depicts the pulse‐encoded representation of the letter ‘A’. The confusion matrix in Figure 7f demonstrates 100% recognition accuracy in the letter classification task, validating the effectiveness and reliability of the proposed optoelectronic encoding approach.

Prior studies have indeed explored synaptic plasticity and ventured into machine learning applications [60]. However, the depth of these investigations remains rather superficial. The present work distinguishes itself through three key advancements. This work achieves high MNIST handwritten digit recognition accuracy alongside exceptional energy efficiency per operation, thereby establishing competitive performance metrics in the field. This work introduces multi‐bit precision capabilities that confer enhanced versatility to the device performance. Most notably, this work pioneers the integration of dynamic response mechanisms with vehicle trajectory analysis, a research direction that remains entirely unexplored in previous literature. For comparative purposes, several neuromorphic computing studies on P3HT‐based optoelectronic synapses are presented (Table S3).

We have systematically addressed these critical practical considerations through comprehensive experimental validation. Regarding long‐term stability, we conducted continuous optical and electrical stress tests on the organic layer, demonstrating stable synaptic behavior over extended operation periods (Figures S5 and S6). For ambient and broadband illumination robustness, we evaluated device performance under white LED lamp conditions, confirming reliable operation across varying spectral compositions (Figures S7 and S8). Furthermore, we quantified device‐to‐device variability by characterizing multiple pixels across the array, achieving acceptable uniformity with coefficient of variation for key metrics, including conductance range and switching voltage (Figure S9).

## 3 Conclusion

In summary, this work introduces a bioinspired optoelectronic neuromorphic array based on ZnO/P3HT ambipolar transistors, which seamlessly integrates sensory perception, in‐memory storage, and computation within a single platform. In the electrical operation mode, the device exhibits excellent endurance exceeding 10^5^ cycles, device‐to‐device non‐uniformity below 2%, and a single‐device energy consumption of 1.2 fJ. The measured nonlinearity values for long‐term potentiation (Np) and long‐term depression (Nd) are 0.003 and 0.004, respectively. When deployed for analog recognition tasks on the MNIST handwritten digit dataset, the system achieves 95.6% accuracy. In the optical operation mode, the device leverages its intrinsic “optical dynamic response” to encode spatiotemporal history directly within individual frames. Furthermore, using an 8‐bit pulse encoding scheme, it attains 100% recognition accuracy for six different two‐color combinations of the letter “A”. Implemented in a 64 × 64 array configuration, the system successfully processes real‐time traffic video at 24 frames per second while maintaining minimal prediction error for next‐frame motion trajectory.

## 4 Methods

### 4.1 Materials

Materials and Reagents: Zinc acetate (Zn(CH_3_COO)_2_, 99.99%), ammonia solution (25%–28%), anhydrous toluene (99.8%), and poly(3‐hexylthiophene‐2,5‐diyl) (P3HT, regioregularity ≥96%, M_n_ ≈ 50 kDa) were used as received. A p‐type silicon wafer with 100 nm thermal SiO_2_ was employed as the substrate.

### 4.2 Preparation of Gate Electrodes and Dielectric Layer

The substrate was sequentially cleaned with acetone, isopropanol, and deionized water under ultrasonication, followed by drying under a nitrogen stream. The silicon wafer was then vapor‐deposited with a gold electrode as the bottom gate.

The cleaned substrate was placed in an atomic layer deposition (ALD) apparatus, with the deposition temperature set at 200°C. Using trimethylaluminum (TMA) and water (H_2_O) as precursors, the pulse durations were 0.2 s each, with 4‐s nitrogen purging cycles repeated 300 times to deposit a 30‐nm‐thick aluminum oxide film. After deposition, the sample was transferred to an annealing furnace. Under a nitrogen atmosphere, it was heated at 5°C/min to 250°C, maintained for 2 h, and then cooled to room temperature to complete the annealing process.

### 4.3 Deposition of ZnO and P3HT Layer

Zinc acetate (0.10 mol) was dissolved in 50 mL deionized water and stirred magnetically at 30°C for 30 min. Ammonia solution was added dropwise until the pH reached 10.5, forming a colorless transparent [Zn(NH_3_)_4_]^2^^+^ complex solution (0.2 mol L^−^^1^). The cleaned substrate was treated with UV‐ozone for 15 min, spin‐coated with the precursor solution at 1500 rpm for 30 s, and immediately transferred to a hotplate. The film was pre‐baked at 60°C for 10 min to volatilize ammonia, forming a Zn(OH)_2_ wet film, then annealed at 180°C in air for 1 h to yield a dense amorphous ZnO (a‐ZnO) layer (∼10 nm thick).

In a nitrogen glovebox, P3HT was dissolved in anhydrous toluene and stirred at 60°C for 4 h to obtain a clear solution (0.8 mg mL^−^^1^). The solution was allowed to stand for 1 h for defoaming. After the ZnO substrate cooled to room temperature, the P3HT solution was spin‐coated at 1500 rpm for 45 s, followed by annealing at 100°C for 10 min to remove residual solvent.

### 4.4 Electrode Fabrication

All evaporation processes were conducted in a high‐vacuum multi‐source beam deposition system at pressures below 5 × 10^−^^4^ Pa. A single‐use stainless steel mask (50 µm thick, laser‐microfabricated) enabled non‐lithographic pattern transfer.

### 4.5 Characterization

Surface morphology and work function mapping were performed using a Bruker Dimension Icon AFM under ambient conditions. Topography imaging was conducted in tapping mode with PPP‐NCHR tips (Nanosenors, *k* ≈ 42 N m^−^^1^, *f* ≈ 320 kHz), a scan rate of 0.8–1 Hz, and 512 × 512 pixel resolution. KPFM measurements employed dual‐pass amplitude‐modulation mode (lift height = 50 nm) with Pt/Ir‐coated tips (SCM‐PIT, Bruker). The contact potential difference (CPD) between the tip and sample was recorded under dark conditions. Electrical characterization was carried out using a Keysight B2902B Precision Source/Measure Unit connected to a micromanipulator probe station. The KPFM system employs a wavelength‐tunable continuous‐wave laser with an output range of 0.1–2 mW cm^−^^2^, which is strictly limited by its design for non‐invasive surface potential mapping and minimal thermal perturbation to the organic active layer. The custom‐built laser system for array‐level experiments integrates multiple laser diodes with beam homogenization optics to achieve uniform illumination across the 16 × 16 pixel area.

### 4.6 Networks

The Tetris pattern recognition was implemented through in situ operations within the array architecture. The vehicle orientation dataset was constructed based on dynamic response mapping from a 16 × 16 scale array. The CNN neural network architecture for recognition was simulated in Python according to hardware backpropagation logic (Equation 1). The vehicle afterimage trajectory was generated by segmenting the entire image into 16 sub‐regions for sequential illumination using the 16 × 16 array, with the subsequent prediction network implemented via a Python‐simulated multilayer perceptron. For the letter recognition task, the encoding was performed through the array, while the readout layer was solved using linear regression (Equation 2).

(1) $$G=\left\{\begin{array}{c} G_{\max},\mathrm{if}\;G+\Delta G>G_{\max}\\ G_{\min},\;\mathrm{if}\;G+\Delta G<G_{\min}\\ G+\Delta G,\;\mathrm{otherwise} \end{array}\right.$$

(2) $$w={(X^{T}X)}^{-1}X^{T}Y$$

## Conflicts of Interest

The authors declare no conflicts of interest.

## Supporting information




**Supporting File**: advs75246‐sup‐0001‐SuppMat.docx.

## Data Availability

The data that support the findings of this study are available from the corresponding author upon reasonable request.
