# Supplementary material for: Ambipolar Organic–Inorganic Heterostructure Transistor Array for Integrated Visual Information Processing
Source: Adv Sci (Weinh). 2026 Apr 10:e75246. Online ahead of print. doi: 10.1002/advs.75246 (PMC13334647; doi:10.1002/advs.75246)
Supplement: Supplementary file 1 — Supporting File: advs75246‐sup‐0001‐SuppMat.docx. [file ADVS-9999-e75246-s001.docx]

Supporting Information

**Ambipolar Organic-Inorganic Heterostructure Transistor Array for Integrated Visual Information Processing**

Wen-Min Zhong, Wenbin Zhang, Yu-Xiang Zeng, JiYu Zhao, Ziqi Jia, Loganathan Veeramuthu, Guanglong Ding, Yan Yan, Meng Zhang, Su-Ting Han, Vellaisamy A. L. Roy, Fengyun Wang,* Chi-Ching Kuo,* and Ye Zhou*

W.-M. Zhong

College of Civil and Transportation Engineering, Shenzhen University, Shenzhen 518060, P. R. China

W.-M. Zhong, W. Zhang, Y.-X. Zeng, Z. Jia, Y. Zhou

Institute for Advanced Study, Shenzhen University, Shenzhen 518060, P. R. China. E-mail: yezhou@szu.edu.cn

J. Zhao

State Key Laboratory of Fine Chemicals, Frontiers Science Center for Smart Materials, Dalian University of Technology, Dalian 116024, P. R. China

L. Veeramuthu, C.-C. Kuo

Institute of Organic and Polymeric Materials, National Taipei University of Technology, Taipei 10608, Taiwan. E-mail: kuocc@mail.ntut.edu.tw

G. Ding, Y. Yan, M. Zhang, Y. Zhou

State Key Laboratory of Radio Frequency Heterogeneous Integration, Shenzhen University, Shenzhen 518060, P. R. China

S.-T. Han

Department of Applied Biology and Chemical Technology, The Hong Kong Polytechnic University, Hung Hom, Hong Kong 999077, P. R. China

V. A. L. Roy

School of Science and Technology, School of Science and Technology, Hong Kong Metropolitan University, Ho Man Tin, Hong Kong 999077, P. R. China

V. A. L. Roy

Hong Kong Metropolitan University Shenzhen Research Institute, Nanshan District, Shenzhen 518000, P. R. China

F. Wang

College of Physics, Qingdao University, Qingdao 266071, P. R. China. E-mail: fywang@qdu.edu.cn

C.-C. Kuo

Advanced Research Center for Green Materials Science and Technology, National Taiwan University, Taipei 10617, Taiwan.

$$G_{LTP}=G_{min}+B(1-e^{-\frac{P}{A}})$$

$$G_{LTD}=G_{max}-B(1-e^{\frac{P-P_{max}}{A}})$$

$$B=\frac{G_{max}-G_{min}}{1-e^{\frac{P_{max}}{A}}}$$

where G_LTP_ and G_LTD_ are the conductance of device for the potential and depression, G_max_ and G_min_ are their maximum and minimum values, P is the pulse number, and P_max_ is its maximum value that can be applied to the device to adjust its conductance. The A value is inversely proportional to nonlinearity of the LTP/LTD curves, and B is a fitting parameter, which is a function of A.


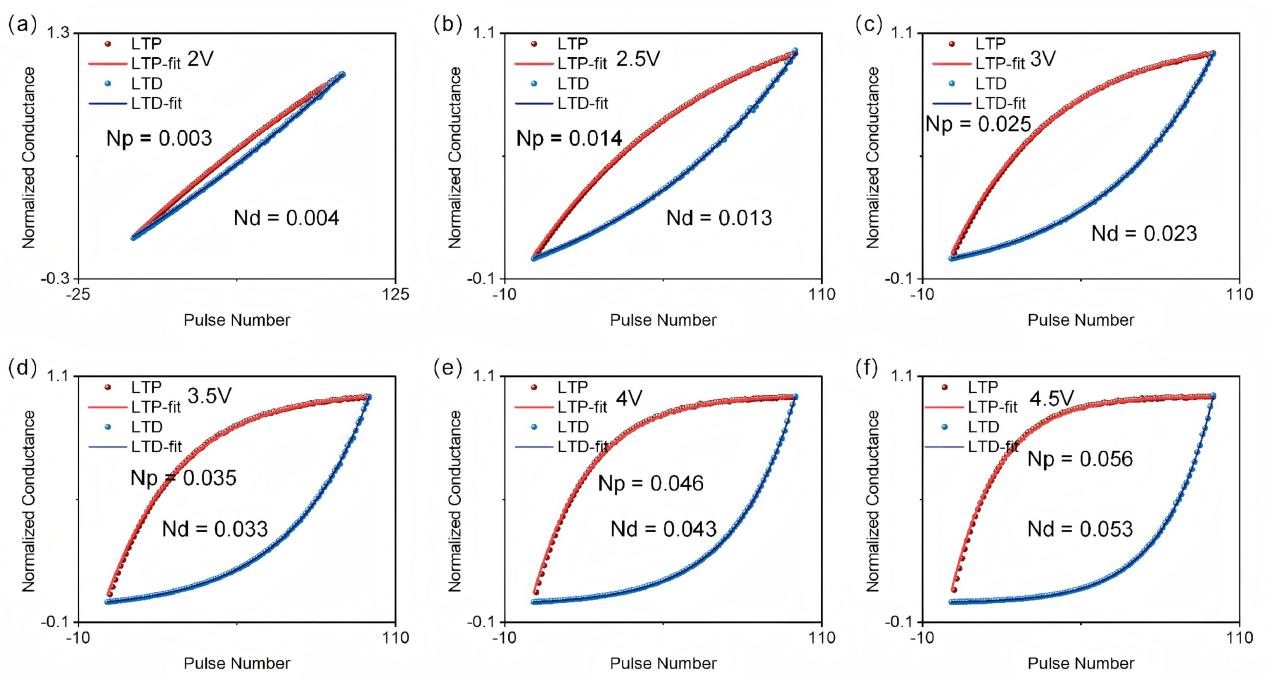


Figure S1. Normalized long-term potentiation (LTP) and long-term depression (LTD) under different programming voltages. Symbols represent experimental data solid lines are nonlinear fits based on the above formula.


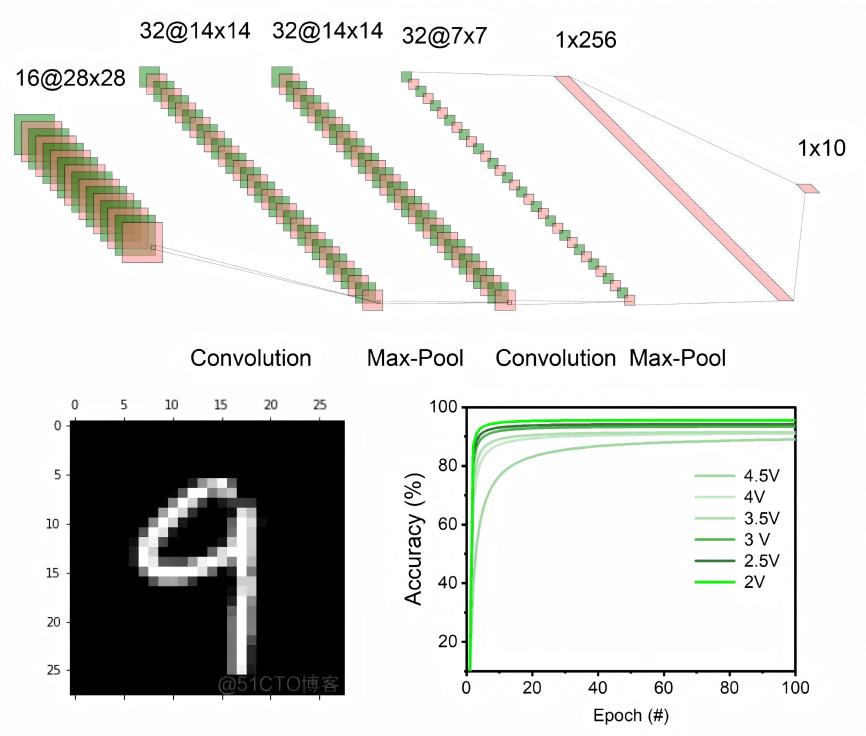


Figure S2. Neuromorphic computational simulation of MNIST dataset in electrical mode.

Table S1: Power consumption of neuromorphic computing devices and accuracy of analog recognition on MNIST handwritten digit dataset.

| Device type | Material | Power | Task | Acc | Ref |
| --- | --- | --- | --- | --- | --- |
| Transistors | PEDOT:PSS | 113 fJ | MNIST | 95.5% | [1] |
| Memristor | GaN | 2.72 pJ | MNIST | 93% | [2] |
| Memristor | LiClO_4_ | <500 fJ | MNIST |  | [3] |
| Transistors | h-BN/Te | 9 fJ | MNIST | 93% | [4] |
| Memristor | Bi_2_O_2_Se | <3.02 pJ | MNIST | 90.6% | [5] |
| Memristor | Ta/Ta_2_O_5_/AlN/graphene | 37 fJ | MNIST |  | [6] |
| Transistors | 2D Te | 110 fJ | MNIST | 88.2% | [7] |
| Memristor | Si:HfO_2_ | 32.65 fJ | MNIST | 96.23 % | [8] |
| Transistor | P3HT/ZnO | 1.2 fJ | MNIST | 95.6% | This work |

[1] S.-K. Lee, Y. W. Cho, J.-S. Lee, Y.-R. Jung, S.-H. Oh, J.-Y. Sun, S. Kim, Y.-C. Joo, Nanofiber Channel Organic Electrochemical Transistors for Low-Power Neuromorphic Computing and Wide-Bandwidth Sensing Platforms, Adv. Sci. 2021, 8, 2001544.

[2] M. Zhou, Y. Zhao, X. Gu, Q. Zhang, J. Zhang, M. Jiang, S. Lu, Light-stimulated low-power artificial synapse based on a single GaN nanowire for neuromorphic computing, Photonics Res. 2023, 11, 1667.

[3] M.T. Sharbati, Y. Du, J. Torres, N. D. Ardolino, M. Yun, F. Xiong, Low-Power, Electrochemically Tunable Graphene Synapses for Neuromorphic Computing, Adv. Mater. 2018, 30, 1802353.

[4] B.You, J. Yoon, Y. Kim, M. Yang, J. Bak, J. Park, U. J. Kim, M. G. Hahm, M. Lee,An extremely low-power-consumption reconfigurable two-dimensional tellurene artificial synapse for bio-inspired wearable edge computing, J. Mater. Chem. C 2024, 12, 6596.

[5] Z. Dong, Q. Hua, J. Xi, Y. Shi, T. Huang, X. Dai, J. Niu, B. Wang, Z. L. Wang, W. Hu, Ultrafast and Low-Power 2D Bi2O2Se Memristors for Neuromorphic Computing Applications, Nano Lett. 2023, 23, 3842.

[6] X. Yan, G. Cao, J. Wang, M. Man, J. Zhao, Z. Zhou, H. Wang, Y. Pei, K. Wang, C. Gao, J. Lou, D. Ren, C. Lu, J. Chen, Memristors based on multilayer graphene electrodes for implementing a low-power neuromorphic electronic synapse, J. Mater. Chem. C 2020, 8, 4926.

[7] J. Yoon, B. You, Y. Kim, J. Bak, M. Yang, J. Park, M. G. Hahm, M. Lee, Environmentally Stable and Reconfigurable Ultralow-Power Two-Dimensional Tellurene Synaptic Transistor for Neuromorphic Edge Computing, ACS Appl. Mater. Interfaces 2023, 15, 18463.

[9] T. Wang, J. Meng, X. Zhou, Y. Liu, Z. He, Q. Han, Q. Li, J. Yu, Z. Li, Y. Liu, H. Zhu, Q. Sun, D. W. Zhang, P. Chen, H. Peng, L. Chen, Reconfigurable neuromorphic memristor network for ultralow-power smart textile electronics, Nature Commun. 2022, 13, 7432.

[10] X. Yan, X. Jia, Y. Zhang, S. Shi, L. Wang, Y. Shao, Y. Sun, S. Sun, Z. Zhao, J. Zhao, J. Sun, Z. Guo, Z. Guan, Z. Zhang, X. Han, J. Chen, A low-power Si:HfO2 ferroelectric tunnel memristor for spiking neural networks, Nano Energy 2023, 107, 108091.


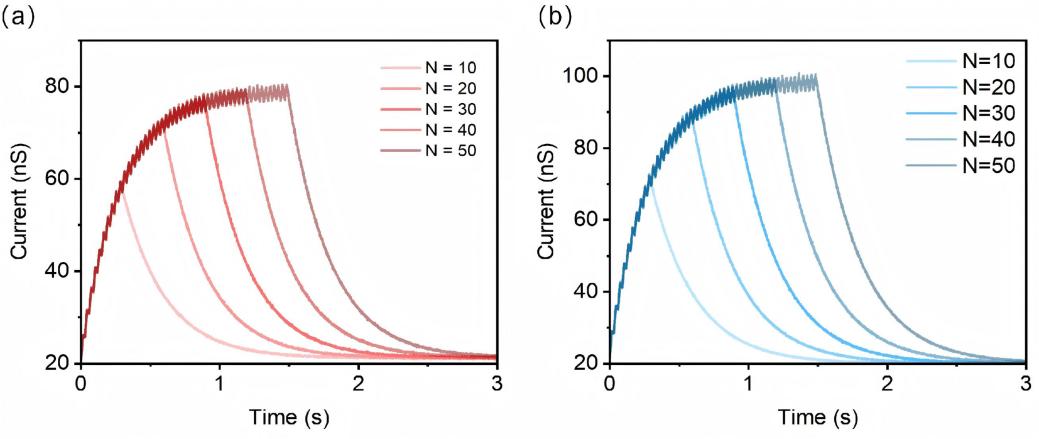


Figure S3. Dynamic response of the device under (a) red and (b) blue illumination as a function of stimulus pulse number.


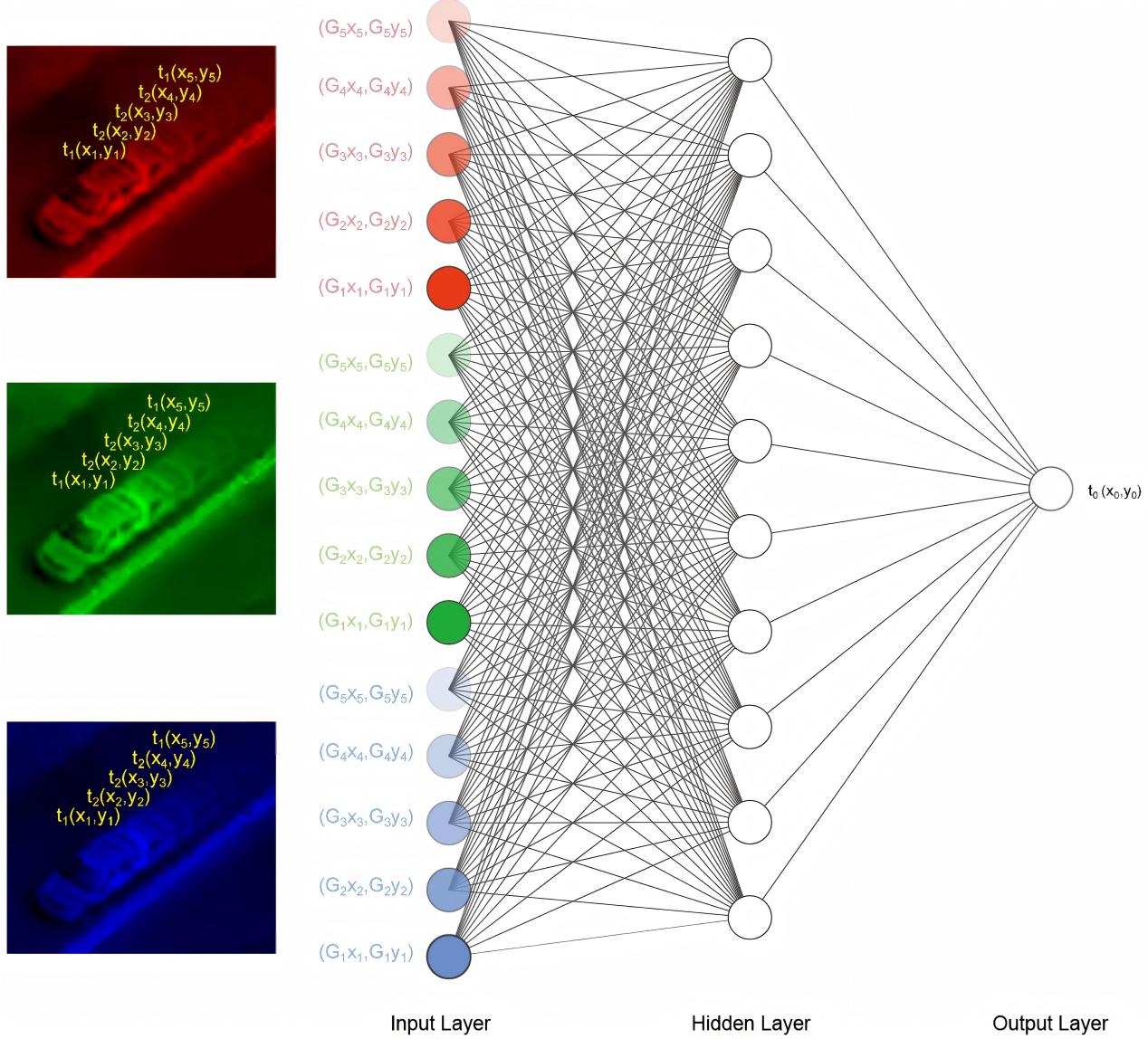


Figure S4. Schematic illustration of path prediction.

Table S2: Part 1 – 8-bit dynamic response

| Code | Red(nA) | Green(nA) | Blue(nA) | Code | Red(nA) | Green(nA) | Blue(nA) |
| --- | --- | --- | --- | --- | --- | --- | --- |
| 00000000 | 20.2 | 20.18 | 20.11 | 10000000 | 21.4 | 22.93 | 21.95 |
| 00000001 | 21.39 | 22.95 | 21.89 | 10000001 | 22.89 | 25.86 | 23.94 |
| 00000010 | 29.42 | 38.97 | 32.65 | 10000010 | 30.42 | 40.83 | 33.91 |
| 00000011 | 30.45 | 40.91 | 33.96 | 10000011 | 31.4 | 42.82 | 35.19 |
| 00000100 | 26.93 | 33.88 | 29.25 | 10000100 | 27.75 | 35.54 | 30.39 |
| 00000101 | 27.85 | 35.88 | 30.57 | 10000101 | 28.7 | 37.59 | 31.67 |
| 00000110 | 33.83 | 47.88 | 38.57 | 10000110 | 34.34 | 48.7 | 39.1 |
| 00000111 | 34.65 | 49.41 | 39.6 | 10000111 | 35.29 | 50.79 | 40.46 |
| 00001000 | 25.04 | 30.06 | 26.7 | 10001000 | 25.97 | 32.09 | 28.02 |
| 00001001 | 26.26 | 32.62 | 28.39 | 10001001 | 27.05 | 34.27 | 29.5 |
| 00001010 | 32.51 | 45.13 | 36.75 | 10001010 | 33.21 | 46.43 | 37.65 |
| 00001011 | 33.63 | 47.34 | 38.25 | 10001011 | 34.09 | 48.18 | 38.75 |
| 00001100 | 30.03 | 40.11 | 33.37 | 10001100 | 30.45 | 41.05 | 34.04 |
| 00001101 | 31.05 | 42.09 | 34.74 | 10001101 | 31.53 | 43.15 | 35.38 |
| 00001110 | 35.9 | 51.89 | 41.3 | 10001110 | 36.23 | 52.68 | 41.76 |
| 00001111 | 36.81 | 53.81 | 42.49 | 10001111 | 37.04 | 54.06 | 42.66 |
| 00010000 | 23.76 | 27.64 | 25.01 | 10010000 | 24.63 | 29.31 | 26.22 |
| 00010001 | 25.11 | 30.25 | 26.87 | 10010001 | 25.77 | 31.6 | 27.65 |
| 00010010 | 31.73 | 43.64 | 35.67 | 10010010 | 32.18 | 44.48 | 36.32 |
| 00010011 | 32.77 | 45.59 | 37.07 | 10010011 | 33.38 | 46.81 | 37.86 |
| 00010100 | 29.09 | 38.22 | 32.14 | 10010100 | 29.67 | 39.4 | 32.92 |
| 00010101 | 30.15 | 40.47 | 33.57 | 10010101 | 30.55 | 41.27 | 34.17 |
| 00010110 | 35.2 | 50.59 | 40.35 | 10010110 | 35.65 | 51.33 | 40.88 |
| 00010111 | 36.15 | 52.41 | 41.59 | 10010111 | 36.43 | 52.98 | 41.95 |
| 00011000 | 27.25 | 34.72 | 29.75 | 10011000 | 27.82 | 35.7 | 30.45 |
| 00011001 | 28.46 | 36.96 | 31.32 | 10011001 | 28.99 | 38.03 | 32.02 |
| 00011010 | 34.24 | 48.47 | 38.97 | 10011010 | 34.42 | 49.03 | 39.29 |
| 00011011 | 34.98 | 50.07 | 40.03 | 10011011 | 35.4 | 50.84 | 40.57 |
| 00011100 | 31.49 | 43.07 | 35.39 | 10011100 | 31.65 | 43.46 | 35.58 |
| 00011101 | 32.41 | 44.88 | 36.55 | 10011101 | 32.52 | 45.27 | 36.81 |
| 00011110 | 36.98 | 53.97 | 42.6 | 10011110 | 37.12 | 54.27 | 42.88 |
| 00011111 | 37.79 | 55.67 | 43.77 | 10011111 | 38.01 | 56.07 | 44 |

Table S2: Part2 – 8-bit dynamic response

| Code | Red(nA) | Green(nA) | Blue(nA) | Code | Red(nA) | Green(nA) | Blue(nA) |
| --- | --- | --- | --- | --- | --- | --- | --- |
| 00100000 | 22.71 | 25.41 | 23.66 | 10100000 | 23.68 | 27.37 | 24.95 |
| 00100001 | 23.99 | 27.95 | 25.26 | 10100001 | 24.86 | 29.88 | 26.49 |
| 00100010 | 31.16 | 42.36 | 34.89 | 10100010 | 31.73 | 43.66 | 35.69 |
| 00100011 | 32.03 | 44.27 | 36.14 | 10100011 | 32.6 | 45.22 | 36.78 |
| 00100100 | 28.64 | 37.43 | 31.58 | 10100100 | 29.05 | 38.09 | 32.05 |
| 00100101 | 29.65 | 39.46 | 32.98 | 10100101 | 30.05 | 40.14 | 33.41 |
| 00100110 | 34.93 | 49.9 | 39.88 | 10100110 | 35.41 | 50.94 | 40.6 |
| 00100111 | 35.78 | 51.54 | 40.97 | 10100111 | 36.17 | 52.42 | 41.64 |
| 00101000 | 26.73 | 33.61 | 29.01 | 10101000 | 27.27 | 34.6 | 29.77 |
| 00101001 | 27.79 | 35.77 | 30.5 | 10101001 | 28.57 | 37.15 | 31.43 |
| 00101010 | 33.7 | 47.56 | 38.34 | 10101010 | 33.99 | 48.02 | 38.66 |
| 00101011 | 34.51 | 49.12 | 39.43 | 10101011 | 34.85 | 49.89 | 39.84 |
| 00101100 | 30.97 | 42.06 | 34.73 | 10101100 | 31.26 | 42.65 | 35.14 |
| 00101101 | 32.07 | 44.11 | 36.08 | 10101101 | 32.2 | 44.62 | 36.37 |
| 00101110 | 36.55 | 53.25 | 42.14 | 10101110 | 36.87 | 53.84 | 42.46 |
| 00101111 | 37.4 | 54.92 | 43.24 | 10101111 | 37.64 | 55.22 | 43.5 |
| 00110000 | 25.34 | 30.74 | 27.15 | 10110000 | 25.67 | 31.59 | 27.7 |
| 00110001 | 26.57 | 33.23 | 28.8 | 10110001 | 27.1 | 34.36 | 29.52 |
| 00110010 | 32.77 | 45.52 | 37.05 | 10110010 | 32.98 | 46.16 | 37.37 |
| 00110011 | 33.96 | 47.93 | 38.61 | 10110011 | 34 | 48.14 | 38.76 |
| 00110100 | 30.15 | 40.51 | 33.64 | 10110100 | 30.49 | 41.17 | 34.09 |
| 00110101 | 31.09 | 42.24 | 34.82 | 10110101 | 31.46 | 42.98 | 35.29 |
| 00110110 | 35.98 | 52.03 | 41.34 | 10110110 | 36.21 | 52.46 | 41.68 |
| 00110111 | 36.83 | 53.62 | 42.42 | 10110111 | 37.03 | 54.22 | 42.79 |
| 00111000 | 28.3 | 36.59 | 31.02 | 10111000 | 28.68 | 37.41 | 31.65 |
| 00111001 | 29.33 | 38.73 | 32.45 | 10111001 | 29.64 | 39.47 | 32.87 |
| 00111010 | 34.56 | 49.29 | 39.47 | 10111010 | 34.97 | 50.03 | 39.93 |
| 00111011 | 35.52 | 51.16 | 40.75 | 10111011 | 35.75 | 51.69 | 41.08 |
| 00111100 | 32.05 | 44.24 | 36.15 | 10111100 | 32.24 | 44.58 | 36.37 |
| 00111101 | 32.91 | 45.96 | 37.29 | 10111101 | 33.1 | 46.4 | 37.59 |
| 00111110 | 37.58 | 55.19 | 43.38 | 10111110 | 37.63 | 55.37 | 43.56 |
| 00111111 | 38.21 | 56.46 | 44.3 | 10111111 | 38.4 | 56.94 | 44.61 |

Table S2: Part3 – 8-bit dynamic response

| Code | Red(nA) | Green(nA) | Blue(nA) | Code | Red(nA) | Green(nA) | Blue(nA) |
| --- | --- | --- | --- | --- | --- | --- | --- |
| 01100000 | 22.08 | 24.15 | 22.74 | 11000000 | 22.82 | 25.76 | 23.84 |
| 01100001 | 23.36 | 26.72 | 24.47 | 11000001 | 24.12 | 28.42 | 25.57 |
| 01100010 | 30.65 | 41.31 | 34.19 | 11000010 | 31.32 | 42.59 | 35.11 |
| 01100011 | 31.6 | 43.24 | 35.42 | 11000011 | 32.15 | 44.36 | 36.2 |
| 01100100 | 28.05 | 36.26 | 30.74 | 11000100 | 28.63 | 37.45 | 31.62 |
| 01100101 | 29.19 | 38.4 | 32.21 | 11000101 | 29.75 | 39.67 | 33.07 |
| 01100110 | 34.74 | 49.48 | 39.65 | 11000110 | 34.9 | 49.99 | 39.96 |
| 01100111 | 35.41 | 51.01 | 40.64 | 11000111 | 35.85 | 51.86 | 41.14 |
| 01101000 | 26.23 | 32.5 | 28.32 | 11001000 | 26.84 | 33.9 | 29.21 |
| 01101001 | 27.36 | 34.94 | 29.85 | 11001001 | 27.84 | 35.85 | 30.59 |
| 01101010 | 33.35 | 46.88 | 37.87 | 11001010 | 33.76 | 47.56 | 38.37 |
| 01101011 | 34.25 | 48.63 | 39.02 | 11001011 | 34.65 | 49.32 | 39.48 |
| 01101100 | 30.68 | 41.51 | 34.34 | 11001100 | 31.01 | 42.02 | 34.62 |
| 01101101 | 31.77 | 43.71 | 35.8 | 11001101 | 32.11 | 44.37 | 36.2 |
| 01101110 | 36.43 | 52.99 | 42 | 11001110 | 36.72 | 53.59 | 42.35 |
| 01101111 | 37.2 | 54.44 | 42.98 | 11001111 | 37.41 | 54.99 | 43.27 |
| 01110000 | 24.86 | 29.82 | 26.51 | 11010000 | 25.37 | 30.8 | 27.15 |
| 01110001 | 26.24 | 32.57 | 28.31 | 11010001 | 26.54 | 33.16 | 28.7 |
| 01110010 | 32.49 | 44.98 | 36.59 | 11010010 | 32.83 | 45.83 | 37.16 |
| 01110011 | 33.53 | 47.04 | 38.06 | 11010011 | 33.82 | 47.81 | 38.52 |
| 01110100 | 29.98 | 40.05 | 33.34 | 11010100 | 30.05 | 40.17 | 33.49 |
| 01110101 | 31.1 | 42.22 | 34.75 | 11010101 | 31.28 | 42.63 | 35.02 |
| 01110110 | 35.71 | 51.63 | 41.08 | 11010110 | 35.97 | 52.07 | 41.34 |
| 01110111 | 36.51 | 53.19 | 42.1 | 11010111 | 36.88 | 53.86 | 42.51 |
| 01111000 | 28.02 | 36.18 | 30.72 | 11011000 | 28.29 | 36.68 | 31.17 |
| 01111001 | 29.13 | 38.28 | 32.13 | 11011001 | 29.52 | 39.09 | 32.65 |
| 01111010 | 34.6 | 49.22 | 39.43 | 11011010 | 34.73 | 49.53 | 39.66 |
| 01111011 | 35.38 | 50.88 | 40.63 | 11011011 | 35.63 | 51.34 | 40.83 |
| 01111100 | 31.84 | 43.82 | 35.84 | 11011100 | 32.16 | 44.39 | 36.28 |
| 01111101 | 32.8 | 45.57 | 37.08 | 11011101 | 33.05 | 46.23 | 37.45 |
| 01111110 | 37.22 | 54.55 | 42.98 | 11011110 | 37.41 | 54.94 | 43.28 |
| 01111111 | 38.04 | 56.06 | 44.02 | 11011111 | 38.16 | 56.48 | 44.33 |

Table S2: Part 4 – 8-bit dynamic response

| Code | Red(nA) | Green(nA) | Blue(nA) | Code | Red(nA) | Green(nA) | Blue(nA) |
| --- | --- | --- | --- | --- | --- | --- | --- |
| 01100000 | 23.93 | 27.99 | 25.25 | 10100000 | 24.52 | 29.2 | 26.07 |
| 01100001 | 25.25 | 30.54 | 26.97 | 10100001 | 25.69 | 31.39 | 27.63 |
| 01100010 | 31.95 | 43.96 | 35.95 | 10100010 | 32.22 | 44.66 | 36.43 |
| 01100011 | 32.71 | 45.58 | 37.02 | 10100011 | 33.15 | 46.37 | 37.56 |
| 01100100 | 29.39 | 38.92 | 32.61 | 10100100 | 29.62 | 39.32 | 32.86 |
| 01100101 | 30.29 | 40.71 | 33.75 | 10100101 | 30.56 | 41.15 | 34.05 |
| 01100110 | 35.33 | 50.73 | 40.43 | 10100110 | 35.57 | 51.16 | 40.73 |
| 01100111 | 36.3 | 52.71 | 41.74 | 10100111 | 36.42 | 53 | 41.93 |
| 01101000 | 27.44 | 35.04 | 30 | 10101000 | 27.75 | 35.52 | 30.37 |
| 01101001 | 28.51 | 37.04 | 31.37 | 10101001 | 28.9 | 37.89 | 31.89 |
| 01101010 | 34.19 | 48.47 | 38.97 | 10101010 | 34.3 | 48.83 | 39.17 |
| 01101011 | 34.82 | 49.72 | 39.84 | 10101011 | 35.2 | 50.47 | 40.3 |
| 01101100 | 31.5 | 43.12 | 35.42 | 10101100 | 31.78 | 43.66 | 35.7 |
| 01101101 | 32.42 | 44.96 | 36.66 | 10101101 | 32.65 | 45.35 | 36.89 |
| 01101110 | 36.97 | 54.05 | 42.67 | 10101110 | 36.95 | 54 | 42.7 |
| 01101111 | 37.68 | 55.54 | 43.71 | 10101111 | 37.82 | 55.77 | 43.8 |
| 01110000 | 26.06 | 32.27 | 28.18 | 10110000 | 26.43 | 33.01 | 28.67 |
| 01110001 | 27.27 | 34.6 | 29.63 | 10110001 | 27.57 | 35.3 | 30.19 |
| 01110010 | 33.21 | 46.56 | 37.66 | 10110010 | 33.38 | 46.99 | 37.93 |
| 01110011 | 34.19 | 48.39 | 38.91 | 10110011 | 34.23 | 48.55 | 39.04 |
| 01110100 | 30.66 | 41.33 | 34.22 | 10110100 | 30.7 | 41.52 | 34.3 |
| 01110101 | 31.56 | 43.31 | 35.56 | 10110101 | 31.79 | 43.72 | 35.78 |
| 01110110 | 36.38 | 52.73 | 41.84 | 10110110 | 36.4 | 52.8 | 41.78 |
| 01110111 | 37.18 | 54.41 | 42.88 | 10110111 | 37.2 | 54.39 | 42.9 |
| 01111000 | 28.73 | 37.62 | 31.68 | 10111000 | 29.06 | 38.31 | 32.14 |
| 01111001 | 29.88 | 39.76 | 33.12 | 10111001 | 30.15 | 40.43 | 33.58 |
| 01111010 | 35.05 | 50.19 | 40.08 | 10111010 | 35.12 | 50.26 | 40.15 |
| 01111011 | 35.97 | 52 | 41.3 | 10111011 | 36 | 51.99 | 41.36 |
| 01111100 | 32.38 | 44.92 | 36.58 | 10111100 | 32.45 | 45.08 | 36.63 |
| 01111101 | 33.2 | 46.53 | 37.69 | 10111101 | 33.53 | 47.05 | 38.05 |
| 01111110 | 37.49 | 55.12 | 43.36 | 10111110 | 37.77 | 55.67 | 43.78 |
| 01111111 | 38.38 | 56.74 | 44.47 | 10111111 | 38.56 | 57.15 | 44.79 |


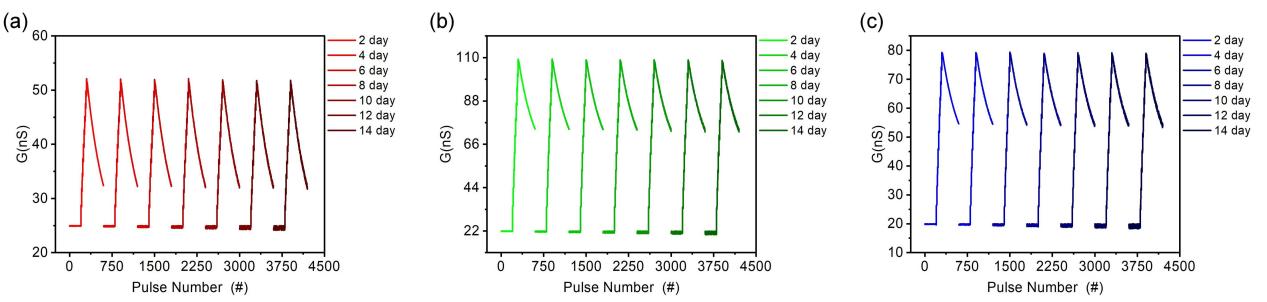


Figure S5. Two-week photodynamic response testing. (a) Dynamic response test under red light stimulation, (b) dynamic response test under green light stimulation, and (c) dynamic response test under blue light stimulation.


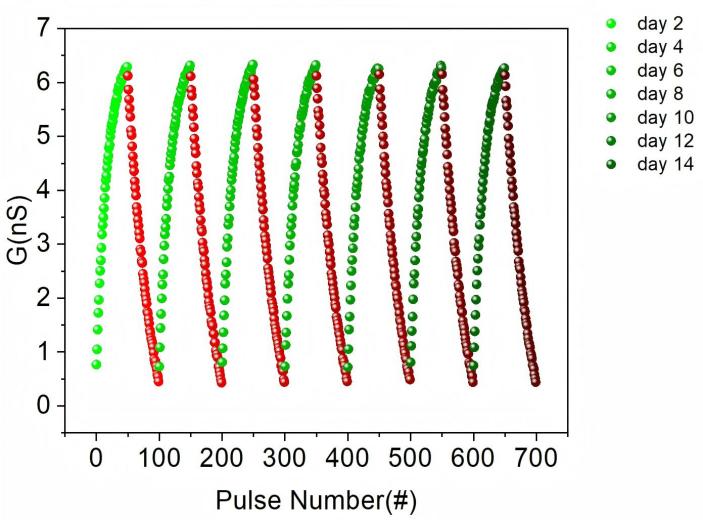


Figure S6. Long-term potential and long-term depression tests with electrical stimulation.


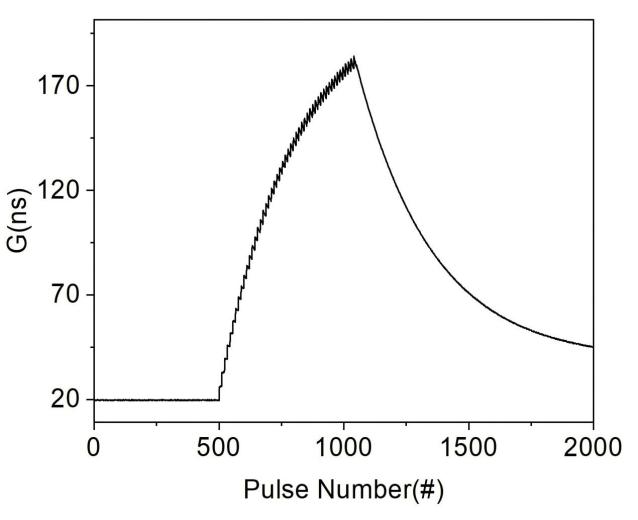


Figure S7. The dynamic response of a wide-bandwidth light source as a pulse.


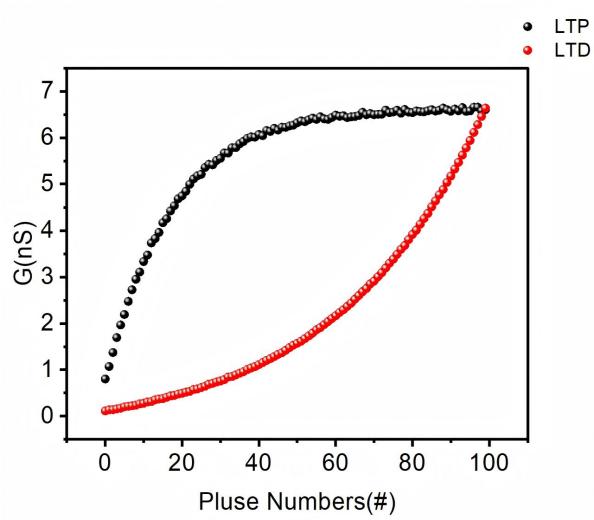


Figure S8. Long-term potential and long-term depression tests achieved through electrical stimulation after broad-band irradiation.


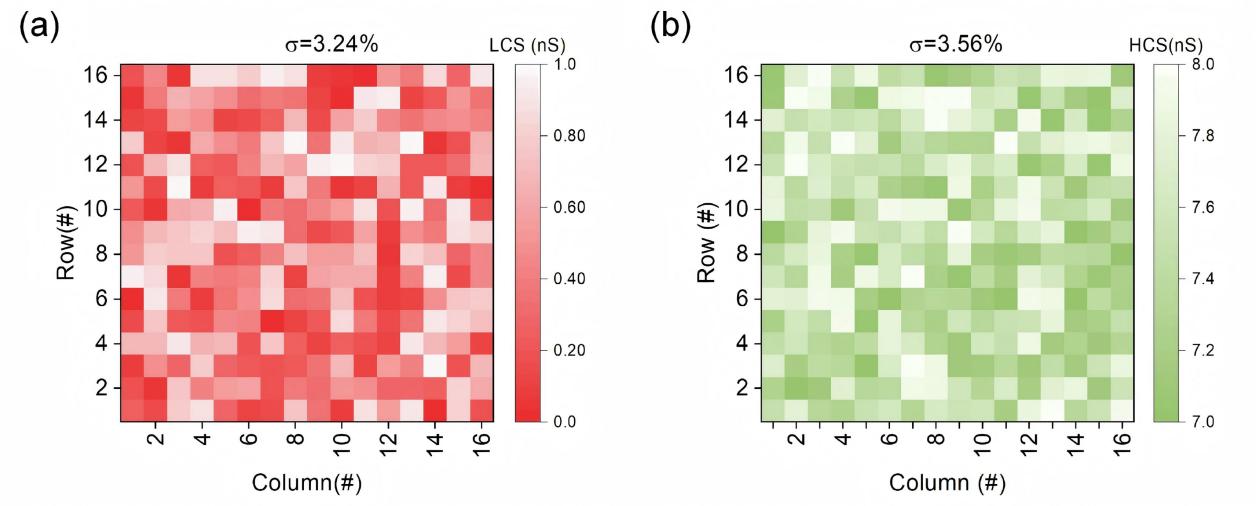


Figure S9. The variability between devices after wide-band irradiation. (a) Variability in low-conductivity state and (b) variability in high-conductivity state.

Table S3: Task performance, power consumption, and bit of three-terminal neuromorphic devices based on P3HT.

| Materials | Task | Power | Bits | ref |
| --- | --- | --- | --- | --- |
| P3HT/GaAs | 94% (color recognition) | ~666.7nJ | 5 bits | [11] |
| P3HT:PCBM | 89.5% (Fashion-MNIST) | ~2.4μJ | / | [12] |
| P3HT/IGT | / | ~5μJ | / | [13] |
| PIDT-BT:P3HT | / | ~32pJ | / | [14] |
| P3HT-*b*-PPI | 94%(MNIST-DIGITS) | ~10pJ | / | [15] |
| P3HT/CYTOP | / | ~27.23nJ | / | [16] |
| P3HT nanoﬁlms | / | ~7.5μJ | / | [17] |
| P3HT-b-PBA, P3HT-b-PS, P3HT-b-PVN to P3HT-b-P2VP | / | ~0.03 to 17.5 fJ | / | [18] |
| Si/P3HT | / | ∼5.2 nJ | / | [19] |
| Si NCs/P3HT | 91.0%(MNIST-DIGITS) | ~10pJ | / | [20] |
| P3HT/PVDF | 92.1%(face recognition) | ~5pJ | / | [21] |
| P3HT:PS | 90.1 %(letter recognition) | ~1.2mJ | 4bits | [22] |
| P3HT-SEBS | / | ~0.48μJ | / | [23] |
| P3HT | / | ~1.6μJ | / | [24] |
| P3HT:APT | / | ~15nJ | / | [25] |
| P3HT:LaF3:Yb/Ho | / | ~4.9nJ | / | [26] |
| P3HT-DPPDTT | / | ~0.48μJ | / | [27] |
| P3HT OECT |  | ~15μJ | / | [28] |
| P3HT-based EGTs | / | ~2.53μJ | / | [29] |
| P3HT(MGFDST) | edge enhancement | ~0.36μJ | / | [30] |
| P3HT/ZnO | 95.6% (MNIST-DIGITS) and Vehicle Trajectory Prediction | 10 fJ | 8bit | This work |

**Reference**

[11] P. Xie, Y. Xu, J. Wang et al. Birdlike broadband neuromorphic visual sensor arrays for fusion imaging. Nat. Commun. 2024, 15, 8298.

[12] K. Chen, H. Hu, I. Song et al. Organic optoelectronic synapse based on photon-modulated electrochemical doping. Nat. Photonics 2023, 17, 629–637.

[13] R. Karimi Azari, Z. Gao, A. Carrière, C. Santato, Exploring response time and synaptic plasticity in P3HT ion-gated transistors for neuromorphic computing: impact of P3HT molecular weight and film thickness. RSC Applied Interfaces 2024, 1, 564.

[14] J. Sun, Y. Liu, Z. Yin, Q. Zheng, High-performance flexible photonic synapse transistors based on a bulk composite film of organic semiconductors with complementary absorption. Acta Chim. Sinica, 2022, 80(7), 936-945.

[15] L. Jiang, L. Yang, Y. Yuan, Q. Zhu, W. Wu, X. Wang, W. Xu, L. Qiu, Flexible Optoelectronic Synapses Based on Conjugated Polymer Blends for Ultra Broadband Spectrum Light Perception. ACS Materials Lett. 2024, 6, 1606.

[16] L. Jiang, C. Xu, X. Wu, X. Zhao, L. Zhang, G. Zhang, X. Wang, L. Qiu, Deep Ultraviolet Light Stimulated Synaptic Transistors Based on Poly(3-hexylthiophene) Ultrathin Films. ACS Appl. Mater. Interfaces 2022, 14, 11718.

[17] J. Qiao, R. Zhang, J. Hu, W.-C. Gao, Y.-D. Zhao, B. Fan, W. Xing, Y.-S. Guan, Freestanding Polymer Nanofilms Based Flexible Synaptic Transistors for Neurological Functions. Adv. Mater. Technol. 2025, 10, e00047.

[18] W. C. Yang, Y. C. Lin, S. Inagaki, H. Shimizu, E. Ercan, L. C. Hsu, C. C. Chueh, T. Higashihara, W. C. Chen, Low‐energy‐consumption and electret‐free photosynaptic transistor utilizing poly (3‐hexylthiophene)‐based conjugated block copolymers. Adv. Sci. 2022, 9, 2105190

[19] Y. Y. Li, Y. Wang, L. Yin, W. Huang, W. B. Peng, Y. Y. Zhu, K. Wang, D. R. Yang, X. D. Pi, Silicon-based inorganic-organic hybrid optoelectronic synaptic devices simulating cross-modal learning. Sci. China Inform. Sci. 2021, 64, 162401.

[20] Y. Wang, Y. Zhu, Y. Li, Y. Zhang, D. Yang, X. Pi, Dual‐modal optoelectronic synaptic devices with versatile synaptic plasticity. Adv. Funct. Mater. 2022, 32, 2107973.

[21] Y. Lee, D. H. Seo, J. S. Lee, J. M. Jeon, H. R. Kim, M. S. Kim, C. Ahn, S.-U. An, J. Choi, H. Kim, C. K. Jeong, H. Lim, D.-H. Kang, Y. M. Song, Revisiting Ferroelectric‐Gated Phototransistors: A Tripartite Synapse‐Inspired Approach to In‐Sensor Image Processing. Adv. Mater. 2026, 38, e03475.

[22] S. H. Chung, J. H. Song, W. W. Lee, H. Yoo, H.-R. Lim, E. K. Lee, Tailored phase separation in P3HT:PS blends for enhanced synaptic performance in organic electrochemical transistors. Synthetic Met. 2025, 314, 117919.

[23] J. Huang, L. Lan, B. Huang, J. Pan, B. Chen, Q. Zhou, H. Su, J. Xu, J. Lin, Y. Liu, Y. Lu, S. Gong, J. Peng, Highly Stretchable Synaptic Transistors Based On Phase-Separated P3HT–SEBS Networks for Adaptative Neuromorphic Computation. ACS Appl. Electron. Mater. 2025, 7, 8236.

[24] J. Gong, T. Li, B. Cao, H. Liu, S. Zhang, M. T. Dove, Photoelectronic synaptic transistors with tuneable synaptic plasticity based on films of P3HT with ordered polymer chains. Mater. Res. Express 2024, 11, 076404.

[25] D.-H. Kim, J. Lee, Y. Kim, H. Yoo, E. K. Lee, Enhanced synaptic behavior of neuromorphic device based on organic electrochemical transistors by adding Aminosilane. Org. Electron. 2024, 131, 107076.

[26] W. Luan, Z. Zhao, H. Li, Y. Zhai, Z. Lv, K. Zhou, S. Xue, M. Zhang, Y. Yan, Y. Cao, G. Ding, S.-T. Han, C.-C. Kuo, Y. Zhou, Near-infrared response organic synaptic transistor for dynamic trace extraction. J. Phys. Chem. Lett. 2024, 15, 8845.

[27] X. Yang, X. Chen, P. Gu, Z. Hu, X. Zhang, Z. Sun, L. Lu, G. Zu, J. Huang, Stretchable semiconducting polymer aerogel transistors for high-performance biosensors and artificial synapses. Biomaterials 2025, 322, 123416.

[28] C. M. Lee, Y. Kim, W. Kim, E. Lee, E. K. Lee, High‐Performance Synaptic Devices Based on Cross‐linked Organic Electrochemical Transistors with Dual Ion Gel. Adv. Funct. Mater. 2025, 35, 2417539.

[29] C. Sun, X. Liu, Q. Yao, Q. Jiang, X. Xia, Y. Shen, X. Ye, H. Tan, R. Gao, X. Zhu, R.-W. Li, A discolorable flexible synaptic transistor for wearable health monitoring. ACS Nano 2024, 18, 515.

[30] J. Wang, Y. Xu, C. Jin, B. Zeng, J. Sun, A flexible multi-gate organic electrochemical synaptic transistor for image processing. Appl. Phys. Lett. 2025, 126, 053501.
